# BIK drives an aggressive breast cancer phenotype through sublethal apoptosis and predicts poor prognosis of ER-positive breast cancer

**DOI:** 10.1038/s41419-020-2654-2

**Published:** 2020-06-11

**Authors:** Vrajesh Pandya, John Maringa Githaka, Namrata Patel, Richard Veldhoen, Judith Hugh, Sambasivarao Damaraju, Todd McMullen, John Mackey, Ing Swie Goping

**Affiliations:** 1 Department of Biochemistry, University of Alberta, Edmonton, AB T6G 2H7 Canada; 2 Department of Lab Medicine and Pathology, University of Alberta, Edmonton, AB T6G 2H7 Canada; 3 Department of Surgery, University of Alberta, Edmonton, AB T6G 2H7 Canada; 4 Department of Oncology, University of Alberta, Edmonton, AB T6G 2H7 Canada

**Keywords:** Targeted therapies, Breast cancer

## Abstract

Apoptosis is fundamental to normal animal development and is the target for many anticancer therapies. Recent studies have explored the consequences of “failed apoptosis” where the apoptotic program is initiated but does not go to completion and does not cause cell death. Nevertheless, this failed apoptosis induces DNA double-strand breaks generating mutations that facilitate tumorigenesis. Whether failed apoptosis is relevant to clinical disease is unknown. BCL-2 interacting killer (BIK) is a stress-induced BH3-only protein that stimulates apoptosis in response to hormone and growth factor deprivation, hypoxia, and genomic stress. It was unclear whether BIK promotes or suppresses tumor survival within the context of breast cancer. We investigated this and show that BIK induces failed apoptosis with limited caspase activation and genomic damage in the absence of extensive cell death. Surviving cells acquire aggressive phenotypes characterized by enrichment of cancer stem-like cells, increased motility and increased clonogenic survival. Furthermore, by examining six independent cohorts of patients (total *n* = 969), we discovered that high BIK mRNA and protein levels predicted clinical relapse of Estrogen receptor (ER)-positive cancers, which account for almost 70% of all breast cancers diagnosed but had no predictive value for hormone receptor-negative (triple-negative) patients. Thus, this study identifies BIK as a biomarker for tumor recurrence of ER-positive patients and provides a potential mechanism whereby failed apoptosis contributes to cancer aggression.

## Introduction

Apoptosis is a cell death program with tumor-suppressor activities. Inhibition of apoptosis causes cancer, and this was first identified in B-cell lymphomas driven by the antiapoptotic protein BCL-2^1,2^. Biological insights into this protein family led to the development of BCL-2 homology domain (BH3) mimetics that inhibit BCL-2 antiapoptotic activities, to initiate the cascade of mitochondrial outer membrane permeabilization (MOMP), caspase activation, and cell death^3,4^. In 2016, ABT-199/Venetoclax attained breakthrough drug designation from the FDA for the treatment of leukemia^5^ and is in clinical testing for other cancers. Hence, apoptosis facilitates tumor cell death and cancer control. Paradoxically, antiapoptotic BCL-2 protein levels are prognostic for favorable outcomes in breast cancer^6–8^ suggesting that attenuated apoptosis limits cancer progression. In line with this, recent studies have shown that low levels of apoptosis are oncogenic^9–11^. Noncancerous cultured cells that were exposed to radiation, chemotherapeutic drugs or the BH3 mimetic ABT-737 became transformed^10–12^. Mechanistically, this “failed apoptosis” stimulated the apoptotic DNases CAD and EndoG that induced DNA double-strand breaks (DSB) leading to genomic instability, and oncogenesis^9–14^. Thus, incompletely executed apoptosis has tumor-promoting consequences in experimental model systems, although the relevance of this process to clinical disease is not clear.

BCL-2 interacting killer (BIK) is a proapoptotic BH3-only member of the BCL-2 family and is prognostic for relapse and decreased overall survival of breast cancer^15^. This suggests that BIK may induce inefficient apoptosis within the context of clinical disease. Here we show that BIK expression in breast cancer cell lines activates caspases and induces genomic damage through caspase-activated DNase (CAD). Despite caspase activation, apoptotic cell death is limited, and the resulting cell progeny show aggressive cell properties. Importantly, BIK expression is induced in response to the blockade of estrogen signaling in ER-positive cells^16–18^ suggesting that BIK may be specifically relevant to ER-positive breast cancer. In line with this, BIK elevation in ER-positive breast cancer patients is associated with increased recurrence and mortality but was not significantly prognostic for hormone receptor-negative patients. Thus BIK-mediated inefficient apoptosis facilitates cellular evolution and identifies a potential new molecular mechanism for recurrence in a subset of breast cancers.

## Results

### BIK can activate the apoptotic pathway while causing minimal cell death

**Fig. 1:**
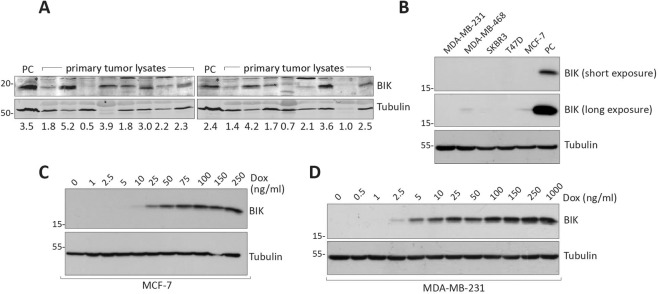
BIK protein levels in primary breast tumors and established cell lines. **a** Western blot and quantitation (numbers at the bottom) of 16 primary breast tumor lysates probed with an anti-BIK antibody. PC indicates positive control—lysates prepared from HeLa cells treated with proteasome inhibitor MG132 known to upregulate BIK protein. Asterisk indicates nonspecific immunoreactive bands. **b** Anti-BIK western blot analysis of cell lysates prepared from five common breast cancer cell lines. Lane indicating PC—lysates prepared from MCF-7 cells transiently transfected with BIK-expressing plasmid were used as a positive control. **c** Western blot analysis of BIK expression in MCF-7 Tet-on cells using the indicated amounts of Dox stimulation for 24 h. **d** Western blot analysis of BIK expression in MDA-MB-231 Tet-on cells using indicated amounts of Dox stimulation for 24 h.

High levels of BIK in breast cancer tumors are prognostic for poor patient outcomes^15^. Since apoptosis is oncogenic when not fully executed in cell-based models^9–12^, we reasoned that BIK accelerated tumor evolution and disease relapse through failed apoptosis. The expression of BIK in tissues and cell lines is variable and is regulated in response to stressors, such as hormone and growth factor withdrawal, hypoxia, and genomic damage^17,19–23^. We therefore first assessed BIK protein levels from snap-frozen primary breast tumor samples and observed that the majority (~80%) of the samples had detectable BIK levels of differing intensity (Fig. 1a). In contrast, established breast cancer cell lines showed barely detectable levels of BIK (Fig. 1b), likely related to the fact that tumor-associated stressors are absent in cell-culture conditions^16,23–25^. In support of this, suppression of estrogen signaling by tamoxifen caused a dose-dependent increase in BIK levels (Supplementary Fig. 1A), confirming the existing ability of cells to induce BIK expression. To investigate BIK autonomous effects on cellular growth properties in the absence of the pleiotropic effects of stressors, we generated doxycycline (Dox)-inducible BIK expression in the breast carcinoma cell lines MCF-7 and MDA-MB-231 (Fig. 1c and d), as representatives of ER-positive and triple-negative breast cancer (TNBC) subtypes, respectively. BIK induction was tightly regulated and titratable in response to Dox, with the expected protein localization to ER-reticular structures (Fig. 1c and d and Supplementary Fig. 1B–D).

**Fig. 2:**
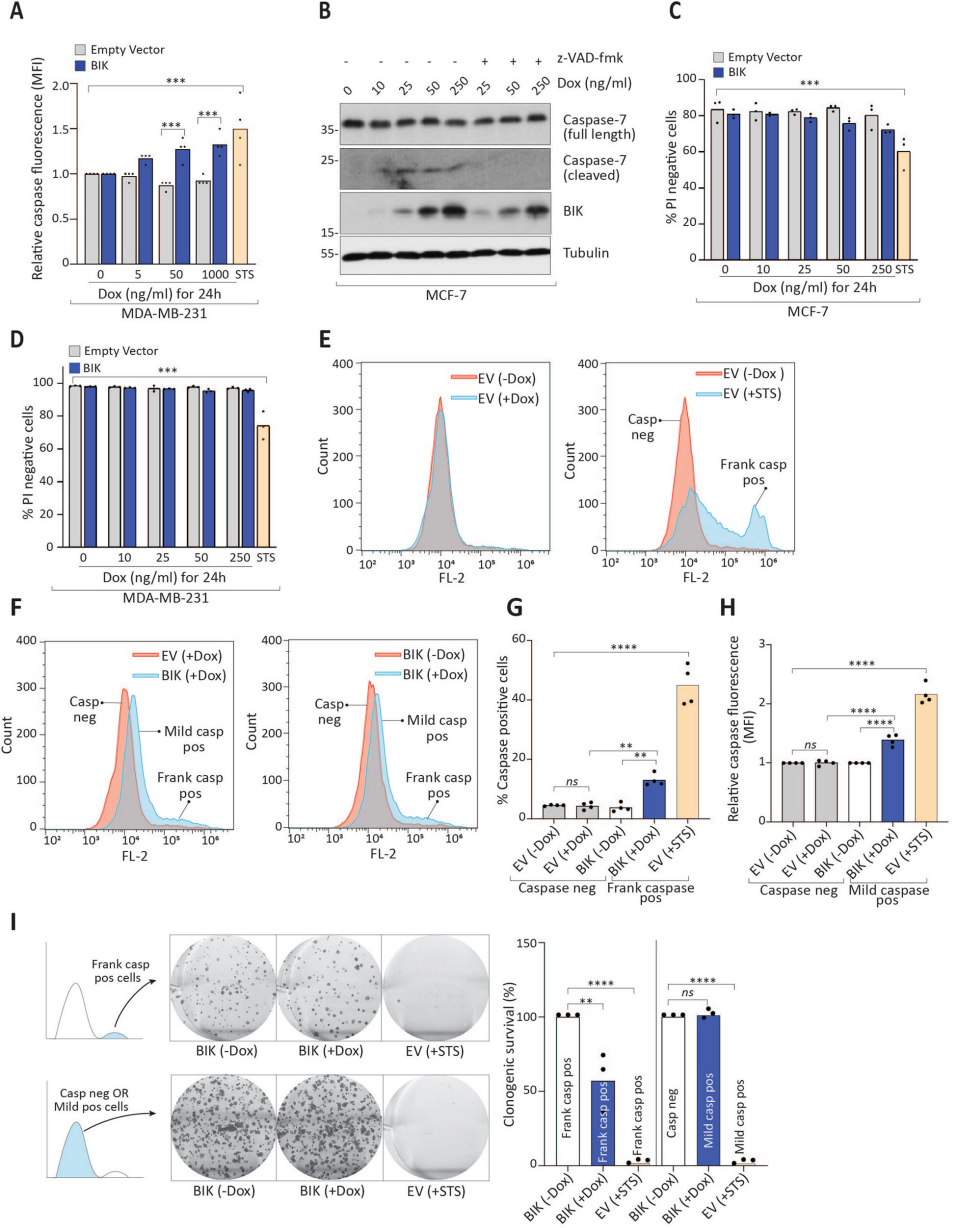
BIK induces sublethal apoptosis with DNA damage. **a** Quantitation of relative mean fluorescence intensity after stimulating MDA-MB-231 Tet-on cells at the indicated doses of Dox for 24 h followed by staining with 10 μM of CaspACE (FITC-VAD-FMK) and flow-cytometric analysis. Four independent experiments were performed and at least 10,000 cells (5000 for staurosporine) were acquired for all treatments. One-way ANOVA followed by Sidak′s post-hoc test was performed to compute significance among groups. **b** Western blot analysis of MCF-7 Tet-on cells expressing BIK at the indicated Dox concentrations in the presence or absence of z-VAD-fmk (10 µM) for 24 h. **c**, **d** MCF-7 and MDA-MB-231 Tet-on cells were, respectively, stimulated with indicated doses of Dox for 24 h followed by staining with propidium iodide and flow-cytometric analysis. At least 10,000 cells were acquired for three independent experiments. One-way ANOVA followed by Sidak′s post-hoc test was performed to compute significance among groups. **e**, **f** Flow-cytometry profiles of MDA-MB-231 Tet-on cells stained with Cell Event Green caspase reporter. Cells were stimulated at the indicated doses of Dox for 24 h followed by staining with 5 μM of Cell Event Green reporter followed by flow-cytometric analysis. Note the rightward shift of the histogram profiles of BIK-expressing cells. 2.5 μM staurosporine was used as a positive control. **g** Bar graph depicting % caspase-positive cells obtained by quantitation of **e**, **f**. Four independent experiments were performed and at least 10,000 cells were acquired for all treatments. One-way ANOVA followed by Sidak′s post-hoc test was performed to compute significance among groups. **h** Bar graph depicting the quantitation of relative mean fluorescence intensity for the main peak of the histograms shown in **e** and **f**. Four independent data points were collected and for all treatments. One-way ANOVA followed by Sidak′s post-hoc test was performed to compute significance among groups. **i** (Left) Representative images of clonogenic survival assay after sorting MDA-MB-231 cells displaying frank or mild caspase activation. Cells were treated with 250 ng/ml of Dox or STS as indicated for 24 h followed by staining with 5 μM of Cell Event Green reporter followed by fluorescence-activated cell sorting. Thousands cells each were collected directly in cell-culture plates from the left or right side cell populations on the histogram by positioning sorting gates in the center of the respective peaks. Colonies were allowed to form for 8 days. Histogram cartoons on the extreme left indicate which part of the flow-cytometry histogram was used to collect cells. (Right) Bar graph depicting % clonogenic survival of frank or mild caspase-positive cells. Three independent experiments were performed. One-way ANOVA followed by Sidak′s post-hoc test was performed to compute significance among groups.

We then characterized BIK-induced apoptotic signaling. BIK expression stimulated subtle yet significant activation of caspases in the majority of MDA-MB-231 cells as measured by the caspase indicator CaspACE (Fig. 2a, and Supplementary Fig. 3). MCF-7 cells are deleted for caspase-3^26^, so we evaluated caspase 7 and observed BIK-induced activation that was caspase dependent, as expected for an executioner caspase (Fig. 2B). Additionally, BIK expression caused a significant loss of mitochondrial potential in ~5% of MDA-MB-231 cells (Supplementary Fig. 2) relative to the untreated or empty vector controls, suggesting mitochondrial effects. Despite caspase activation, there was no significant cell death in either cell-line model as assessed by vital dye uptake for up to 48 h after BIK induction (Fig. 2c, d and Supplementary Fig. 4A and B).

Next, we interrogated the extent of BIK-induced caspase activation and its impact on the long-term survival of cells. We expressed BIK followed by staining with the active caspase reporter dye (Cell Event Green) to quantitate caspase-3/7 positive cells. Intriguingly, the entire population of BIK-expressing cells activated caspases, with ~10% of cells showing ~30-fold frank caspase activation while the remaining 90% had mild but statistically significant 1.4-fold activation of caspases (Fig. 2e–h). Thus, BIK expression produced two distinct cell populations with either mild or frank levels of caspase activation. We separated these two populations by fluorescence-activated cell sorting (FACS) and assessed cellular clonogenic potential with colony-formation assays. Of the frankly positive cell population from BIK-expressing cells, clonogenic potential was reduced by ~50% (Fig. 2i, top panel). This was in contrast to frankly caspase-positive cells from staurosporine treatment, which formed no colonies. Thus, cells could tolerate high levels of BIK-induced caspase activity, but not staurosporine-induced caspase activation. Intriguingly, cells with BIK-induced mild caspase activity showed robust clonogenic potential, while staurosporine-treated cells with similar mild caspase activation did not survive (Fig. 2i, bottom panel). Therefore, despite BIK-mediated caspase activation, cells survived and formed colonies. To confirm this, we also assessed the proliferative capacity of cultured cells for up to 8 days with continued BIK induction and found that cells proliferated with similar kinetics as control cells (Supplementary Fig, 4C–G). Thus, BIK triggered caspase activation, although cells remained viable, maintained clonogenic potential, and ability to proliferate, demonstrating that BIK induced failed or sublethal apoptosis in the majority of cells.

**Fig. 3:**
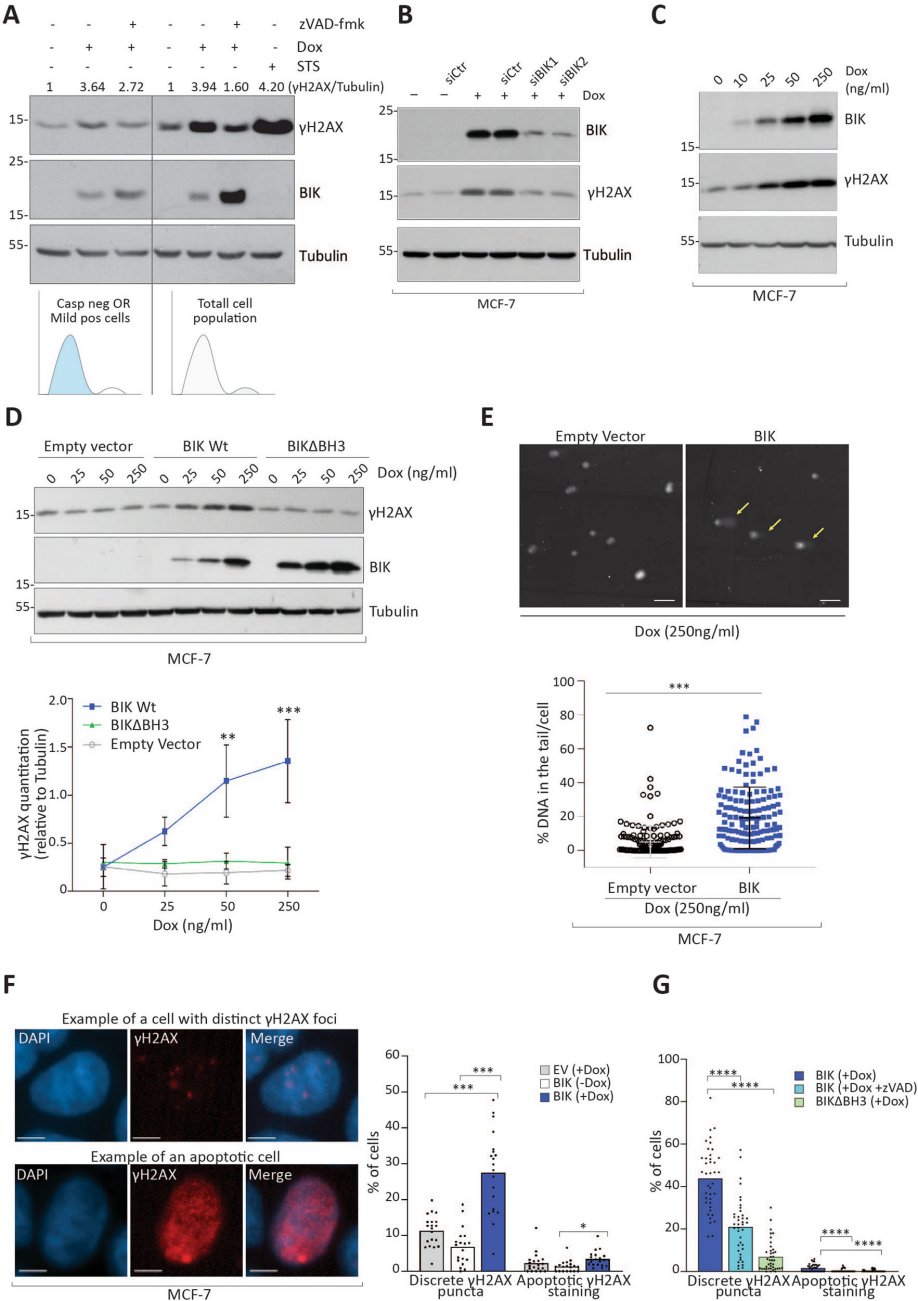
BIK induces discrete foci of DNA damage. **a** Western blot analysis after sorting MDA-MB-231 cells displaying mild caspase activation (left) or total cell population. Cells were treated with 250 ng/ml of Dox or STS (2.5 μM) as indicated for 24 h followed by staining with 5 μM of Cell Event Green reporter followed by fluorescence-activated cell sorting. To obtain cells with mild caspase activation, 100,000 cells were collected from the left side cell populations on the FACS histogram by positioning the sorting gate in the center of the peak. For total cell population analysis (right), cells grown and treated in cell-culture plates were directly collected by trypsinization. Floaters were collected in both cases. Cell lysates were prepared in RIPA buffer and 20 μg total protein was resolved on a 12% SDS-PAGE followed by western blotting with the indicated antibodies. Histogram cartoons at the bottom indicate which part of the flow-cytometry histogram was used to collect cells. **b** Western blot showing siRNA mediated knockdown of BIK expression reduces γH2AX formation. MCF-7 Tet-on cells were pretransfected with either scrambled or two BIK specific siRNAs, and 24 h later stimulated with 50 ng/ml Dox. **c** Western blot analysis of BIK expression and γH2AX formation in MCF-7 Tet-on cells using indicated amounts of Dox. **d** Top: Western blot analysis of MCF-7 Tet-on cells expressing either Empty vector, Wt BIK or BIKΔBH3 at the indicated Dox induction for 24 h. Bottom: Densitometric quantitation of three independent western blots was performed using ImageJ. Error bars represent SD. One-way ANOVA followed by Tukey′s post-hoc test was performed to compute significance among groups. **e** Top: Representative images of comets formed by Empty vector or BIK-expressing MCF-7 cells at the indicated Dox stimulation for 24 h. Bottom: % DNA in the comet tail per nuclei was quantitated using CaspLab comet analysis program. A total of 150 nuclei from three independent experiments were analyzed. An unpaired two-tailed *t*-test was performed to determine the p-value. Scale bar 100 µm. **f** Left: Representative images of γH2AX immunofluorescence analysis of BIK-expressing cells. Right: Quantitation of cells with discrete γH2AX puncta representing DNA damage or diffuse staining representing apoptotic morphology. *n* = 19–20 frames of images with 2569 nuclei for BIK (−Dox), 1652 for BIK (+Dox) and 1693 for Empty vector (+Dox) were analyzed from two biological replicates. One-way ANOVA followed by Sidak′s post-hoc test was performed to compute significance among groups. Scale bar 5 µm. **g** Quantitation of cells with discrete γH2AX puncta representing DNA damage or diffuse staining representing apoptotic morphology. *n* = 40 frames of images with 2946 nuclei for BIK (+Dox), 3484 for BIK (+Dox +z-VAD-fmk), and 3804 for BIK∆BH3 (+Dox) were analyzed from four biological replicates. One-way ANOVA followed by Sidak′s post-hoc test was performed to compute significance among groups.

Failed apoptosis stimulates nuclease-dependent DNA fragmentation leading to mutation accumulation and transformation^10–12^. We therefore investigated whether BIK induced DNA fragmentation. We measured phosphorylation of the DNA double-strand break (DSB) marker histone H2AX-Ser139 (γH2AX) in the total cell population as well as in cells with mild caspase activation as detected by Cell Event Green staining followed by FACS-based cell sorting (Fig. 3a). BIK expression increased γH2AX phosphorylation in the total cell population, which was inhibited by the pan-caspase inhibitor z-VAD-fmk (Fig. 3a, right side panel). Intriguingly, BIK-induced mild caspase activation also triggered caspase-dependent γH2AX phosphorylation (Fig. 3a, left side panel) although to a smaller extent. This observation combined with the colony-formation results (Fig. 2i) suggested that the majority of BIK-expressing cells exhibited caspase-dependent DNA damage and survived. Additionally, through siRNA mediated silencing we confirmed that DNA damage in BIK-expressing cells was specific to BIK (Fig. 3b) and increased in a BIK dose-dependent manner (Fig. 3c). BIK resides at the endoplasmic reticulum where it stimulates Ca^2+^ release^27,28^. BIK-stimulated mitochondrial calcium overload may result in reactive oxygen species (ROS) production, which could also generate DSBs. To estimate the contribution of ROS-induced DNA damage in BIK-expressing cells, we first measured ROS levels in individual cells. BIK expression did not increase levels of ROS (Supplementary Fig. 5A), and BIK-induced γH2AX was unaffected by treatment with the ROS scavenger N-acetylcysteine (NAC) (Supplementary Fig. 5B), indicating that ROS was not an effector of BIK-induced DNA damage. Instead, DNA damage was dependent on the BIK–BH3 domain indicative of the classic mitochondrial apoptotic pathway (Fig. 3d). In support of this, DNA damage diminished in the presence of the pan-caspase inhibitor z-VAD-fmk (Supplementary Fig. 5B). This caspase-dependency suggested that Caspase Activated DNase (CAD) contributed to DNA damage and indeed its silencing reduced γH2AX levels (Supplementary Fig. 5C). Finally, to confirm that γH2AX positivity was indeed due to DNA damage, we performed single-cell electrophoresis assays (comet assay) and observed significantly increased levels of genomic DNA cleavage in BIK-expressing cells (Fig. 3e and Supplementary Fig. 5D). Together these data demonstrated that BIK stimulated the mitochondrial apoptotic program that activated caspases and CAD to induce DNA DSBs.

Next, we tested whether the γH2AX positivity was a function of large scale apoptotic DNA cleavage^29^ or discrete DSBs per cells. We analyzed the subcellular morphology of γH2AX-staining and detected less than 5% of the cells with bright, diffuse γH2AX nuclear staining characteristic of advanced apoptosis (Fig. 3f), confirming that BIK induced minimal cell death (Fig. 2c). Instead, nearly one-third of BIK-expressing cells showed γH2AX staining in discrete puncta indicative of limited DSB (Fig. 3f). BIK-mediated discrete γH2AX positivity was dependent on caspase activity, as treatment with the caspase inhibitor z-VAD-fmk or expression of an apoptosis-defective BIK∆BH3 mutant^30^ significantly reduced the numbers of γH2AX puncta (Fig. 3g). Importantly, despite the generation of DNA damage, cells continued to double (Supplementary Fig. 4C–G), suggesting that DNA DSBs were resolved sufficiently to support cell proliferation and expansion.

### BIK induces heritable changes to cell progeny

**Fig. 4:**
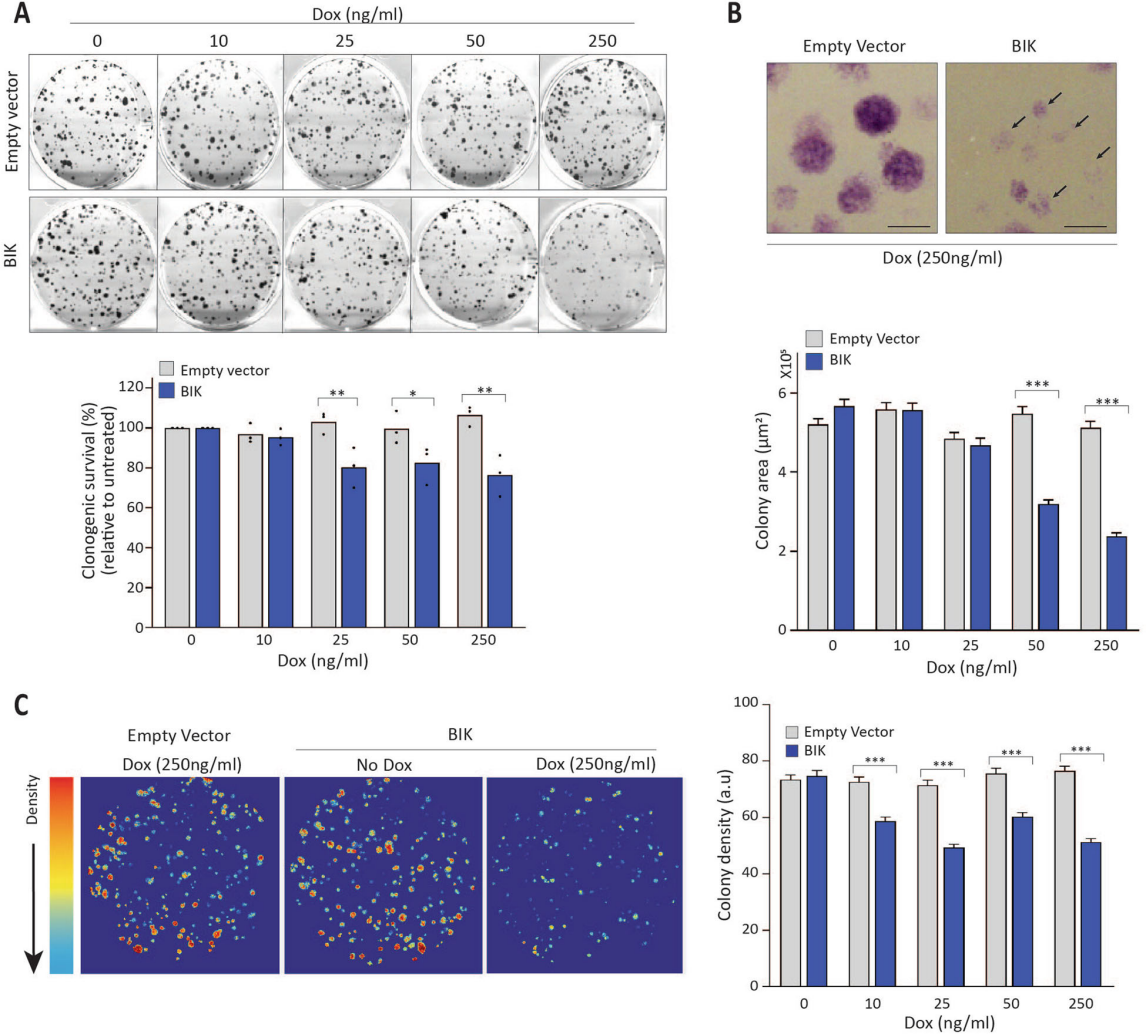
Clonogenic survival assay of MCF-7 cells induced to express BIK. **a** Top: Representative images of clonogenic survival assay performed for Empty vector or BIK-expressing MCF-7 Tet-on cells on continuous Dox stimulation at the indicated Dox concentrations over 11 days. Bottom: Bar graph depicting % clonogenic survival relative to untreated. One-way ANOVA followed by Sidak′s post-hoc test was performed to compute significance among groups. **b** Top: Representative images of colonies formed by MCF-7 Tet-on Empty vector or BIK-expressing cells at 250 ng/ml Dox stimulation. Arrows show colonies with frail morphology. Scale bar 1 mm. Bottom: Colony area was calculated for at least 350 colonies from each group from three different experiments. Error bars represent SEM. One-way ANOVA followed by Sidak′s post-hoc test was performed to compute significance among groups. **c** Left: Representative images depicting cellular density of colonies formed by MCF-7 Tet-on Empty vector or BIK-expressing cells. Red areas indicate high density whereas blue areas indicate low density. Right: Bar graph depicting quantitation of colony density. At least 350 colonies were analyzed from three independent experiments. Error bars indicate SEM. One-way ANOVA followed by Sidak′s post-hoc test was performed to compute significance among groups.

**Fig. 5:**
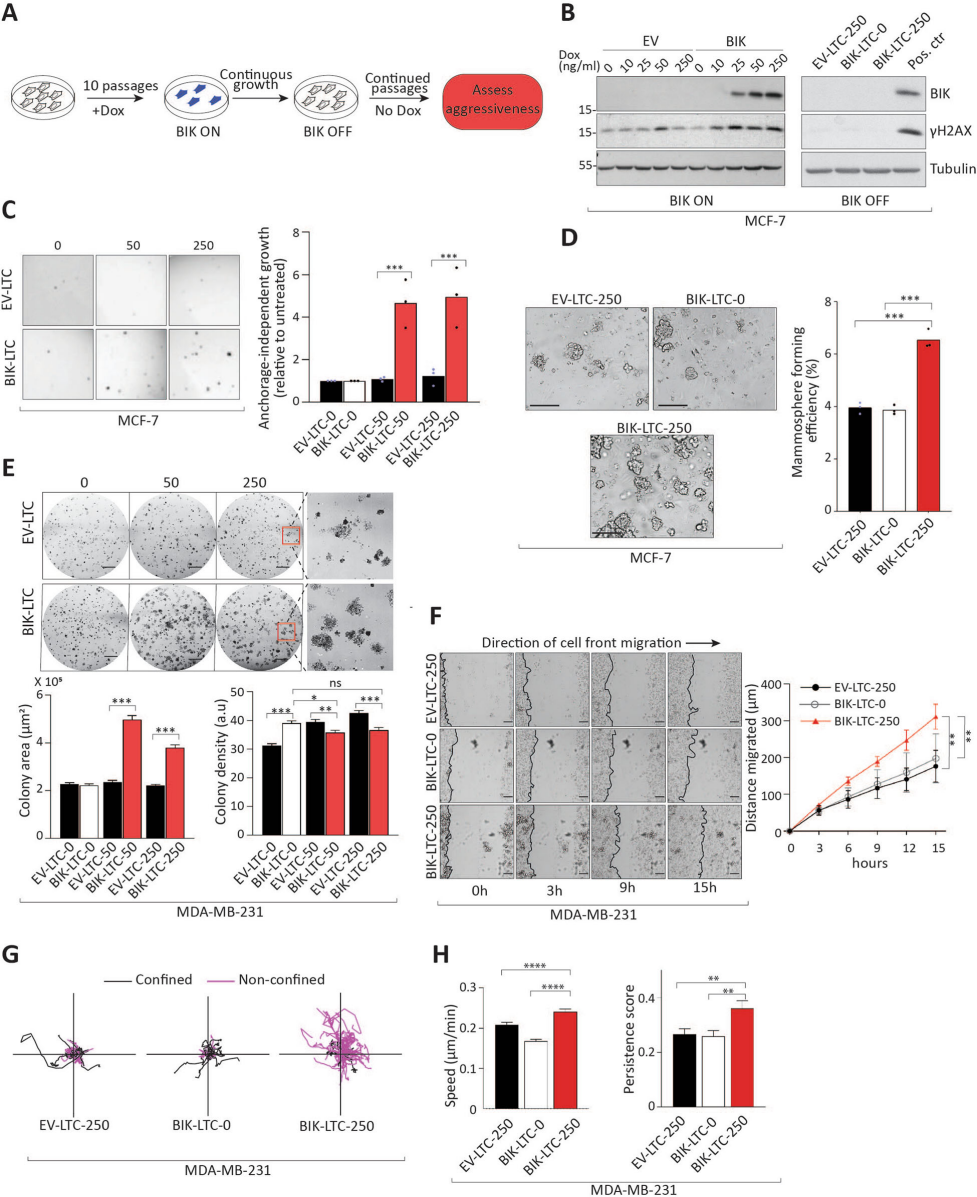
Long-term BIK expression promotes aggressive cell phenotypes. **a** Experimental scheme depicting the generation of “LTC” cells. **b** Left: Western blot analysis performed for MCF-7 Tet-on cells after 10 passages in Dox showing the persistence of BIK expression and DNA damage. Right: Western blot analysis showing BIK expression turned off and DNA damage resolved after Dox withdrawal. Cell lysates made from cells expressing BIK were used as a positive control for anti-BIK and -γH2AX antibodies. **c** Left: Representative images depicting the anchorage-independent growth of MCF-7 LTC cell lines. Right: Quantitation of the fold changes in the number of soft-agar colonies relative to control. Three independent experiments were performed. One-way ANOVA followed by Sidak′s post-hoc test was performed to compute significance among groups. **d** Left: Representative images from mammosphere formation assay performed with MCF-7 LTC cell lines. Mammosphere-forming efficiency (MFE) was calculated after 12 days in culture. Scale bar 250 µm. Right: Bar graph depicting quantitation of the MFE from three independent experiments. One-way ANOVA followed by Sidak′s post-hoc test was performed to compute significance among groups. **e** Top: Representative images from colony-formation assay performed for MDA-MB-231 LTC cells. Scale bar 5 mm. The satellite images show a magnified view of the colonies. Bottom Left: Colony area was calculated for at least 350 colonies from each group from three different experiments. Error bars represent SEM. One-way ANOVA followed by Sidak′s post-hoc test was performed to compute significance among groups. Bottom Right: Colony density was calculated for at least 350 colonies from each group from three different experiments. Error bars represent SEM. One-way ANOVA followed by Sidak′s post-hoc test was performed to compute significance among groups. **f** Left: Representative images at the indicated time-points from the collective cell migration assay performed for MDA-MB-231 LTC cells. Right: Quantitation of the movement of the cell-front over 15 h. A total of nine positions from three independent experiments were analyzed for each group. Error bars represent SD. Linear regression analysis was performed to calculate differences between groups. Scale bar 100 µm. **g** Rose plots depicting the spread of cell movements of the MDA-MB-231 LTC cells. **h** Speed (left) and persistence (right) for MDA-MB-231 LTC cells were calculated by taking the average speed of cells over 24 h. At least 57 tracks were analyzed from three independent experiments. Error bars represent SEM. One-way ANOVA followed by Sidak′s post-hoc test was performed to compute significance among groups.

To examine the consequences of BIK-induced sublethal apoptosis over multiple cell generations, we plated MCF-7 and MDA-MB-231 cells at single-cell density and characterized resulting cell colonies in response to continual BIK expression. The majority of BIK-expressing cells generated colonies with reduced efficiency, indicating defective clonogenic potential (Fig. 4a and Supplementary Fig. 6A). Additionally, BIK-expressing colonies displayed “frail” morphology with significantly reduced colony area (Fig. 4b, and Supplementary Fig. 6B) and density (Fig. 4c and Supplementary Fig. 6C), highlighting differential growth phenotypes. Thus, BIK expression altered growth properties over multiple cell generations. One mechanism whereby sublethal apoptosis induces phenotypic changes is via genomic instability and mutation accumulation that is inherited by cell progeny^9^. To determine whether BIK-induced phenotypic changes were heritable, we expressed BIK continuously for 10 passages, then removed Dox and maintained cells in the absence of Dox/BIK (Fig. 5a). BIK protein expression and subsequent DNA damage persisted for the duration of Dox treatment (Fig. 5b; BIK ON) and became undetectable after the removal of Dox (Fig. 5b; BIK OFF and Supplementary Fig. 7A). This indicated persistent DNA damage in cells grown in the presence of BIK that was diminished in daughter cells following termination of BIK expression. We refer to the progeny of these long-term Dox-treated cells with the annotation “LTC” (for Long Term Culture) and Dox-concentration during treatment. For example, BIK-LTC-250 signifies that BIK expression was induced for 10 passages in 250 ng/mL Dox after which it was maintained in the absence of Dox. These cells were cultured for at least five passages in the absence of Dox/BIK to assess stable changes induced by this “transient” BIK expression. We first tested whether these cells survived 10 passages with high levels of BIK due to a compensatory elevation of antiapoptotic proteins (Supplementary Fig. 8A). BCL-2, BCL-XL, or MCL-1 protein levels were not elevated, and GRP78, which is a specific inhibitor of BIK^31,32^ was also not elevated in the BIK-LTC cell lines. This observation was consistent with patient tumor samples whereby antiapoptotic gene expression and *BIK* gene expression did not correlate^15^.

We next assayed whether long-term BIK expression induced aggressive growth characteristics. We interrogated anchorage-independent growth and stem-like properties that are associated with therapeutic resistance in breast and colon cancer models^33,34^. When prevented from attaching to a solid substratum, MCF-7 BIK-LTC-250 cells formed ~5 times more (Fig. 5c) and MDA-MD-231 BIK-LTC-250 cells formed ~3.5 times more anchorage-independent colonies than their controls (Supplementary Fig. 10A), indicative of an aggressive phenotype. Mammosphere-forming assays that involve cell culture in low-attachment conditions in the absence of serum assess the stem-like properties of cells^35,36^. BIK-LTC-250 cell lines derived from both MCF-7 and MDA-MB-231 cells had ~1.7 times increased numbers of mammosphere colonies than their control EV-LTC-250 or BIK-LTC-0 cell lines, indicating that BIK-treatment increased the proportion of cancer stem-like cells (Fig. 5d and Supplementary Fig. 10B). We measured the area of these mammospheres, which is associated with tumor-initiating potential^37^. Interestingly, the mammospheres formed by MCF-7 BIK-LTC-250 had increased area (Supplementary Fig. 8B), whereas MDA-MB-231 BIK-LTC-250 mammosphere area was unchanged (Supplementary Fig. 10B). Departure from the spherical mammosphere morphology is associated with aggressive phenotypes^38–41^. The mammospheres formed by both MCF-7 and MDA-MB-231 BIK-LTC-250 cells were more irregularly shaped and less spherical compared to their EV-LTC-250 cells as indicated by the isoperimetric quotient that is a measure of circularity (Supplementary Figs. 8B. 10B). Thus, early but nonpersistent BIK expression generated cells with increased anchorage-independent growth and with increased stem-like characteristics indicating that BIK imparted heritable changes in progeny (Supplementary Table 1).

We further characterized if BIK expression altered clonogenic potential in standard 2-dimensional culture conditions. Indeed, MCF-7 BIK-LTC-250 cells had significantly increased colony numbers with unchanged colony area and cell density (Supplementary Fig. 8C and D), consistent with an increased proportion of stem-like cells. Furthermore, we interrogated if BIK-induced aggressiveness required the apoptotic BH3 domain and was caspase-dependent. We therefore generated LTC lines expressing the BIK∆BH3 mutant or cultured wild-type BIK-expressing cells in the presence of z-VAD-fmk for 10 passages (Supplementary Fig. 7B, left side panel, BIK ON). BIK and BIK∆BH3 expression persisted through 10 passages, and as expected, the amount of γH2AX remained lower than the basal γH2AX observed after BIK expression was turned off (Supplementary Fig. 7B, left side panel, BIK OFF). We then assessed the clonogenic potential of the LTC cell lines BIK∆BH3-LTC-250 and BIK-LTC-250-z-VAD relative to BIK-LTC-0 and BIK-LTC-250 cell lines. The clonogenic potentials of BIK∆BH3-LTC-250 or BIK-LTC-250-z-VAD cell lines were not significantly different compared to the negative control BIK-LTC-0 cell line (Supplementary Fig. 8E). In contrast, BIK-LTC-250 cells formed increased numbers of colonies compared to control BIK-LTC-0, BIK∆BH3-LTC-250, and BIK-LTC-250-z-VAD cell lines (Supplementary Fig. 8E). Combined with our previous results, these observations demonstrated that the BH3 domain of BIK and caspase activation are required for BIK-initiated sublethal apoptosis, increased DNA damage and subsequent cancer aggression. While MDA-MB-231 BIK-LTC-250 clonogenic survival was not different than control EV-LTC-250 or BIK-LTC-0 cell lines (Fig. 5e and Supplementary Fig. 10C), BIK expression did induce significant differences in colony morphology. MDA-MB-231 BIK-LTC-250 cells formed colonies with a ~2-fold increase in the colony area and ~1.2-fold reduction in the colony density (Fig. 5e), suggestive of decreased cell adhesion and increased migration. In order to assess the migratory properties of the LTC cells, we tested collective and individual cell migration by wound healing and single-cell tracking assays, respectively. Collective cell migration of the MDA-MB-231 BIK-LTC-250 cells was significantly elevated (Fig. 5f). Furthermore, we investigated if this increase in the migratory properties was dependent on caspase activation. We cultured BIK-expressing MDA-MB-231 cells in the presence of pan-caspase inhibitor z-VAD-fmk for 10 passages (Supplementary Fig. 7B, right side panel, BIK ON). While BIK expression persisted through 10 passages, the amount of γH2AX remained similar to basal γH2AX observed in this cell line after BIK expression was turned off (Supplementary Fig. 7B, right side panel, BIK OFF), confirming that z-VAD-fmk had effectively blocked BIK-mediated caspase-dependent γH2AX production in this cell line. Assessment of collective migration revealed that BIK-LTC-250 cells migrated faster than BIK-LTC-0 and BIK-LTC-250-z-VAD cells (Supplementary Fig. 10D), indicating that evolution of increased cell motility required BIK and caspases. Furthermore, when analyzed at the single-cell level, MDA-MB-231 BIK-LTC-250 cells migrated with higher speed and persistence (the ability of cells to maintain a specific direction of motion) (Fig. 5g and h). The MCF-7 BIK-LTC-250 cell line did not show increased collective or single-cell migration compared to control cells (Supplementary Fig. 9A and B), consistent with an unaltered colony morphology. Thus, BIK expression increased the migratory properties of MDA-MB-231 but not MCF-7 progeny, indicating that BIK altered molecular pathways in a cell-line-specific manner, which facilitated the evolution of differential cell phenotypes.

Taken together, these data are consistent with the proposition that BIK can facilitate heritable changes to pathways that increase the proportion of cancer stem-like cells, anchorage-independent growth, cell migration as well as colony-formation ability in the surviving cell population.

### High *BIK* mRNA and protein levels predict unfavorable outcomes in ER-positive breast cancer patients

**Fig. 6:**
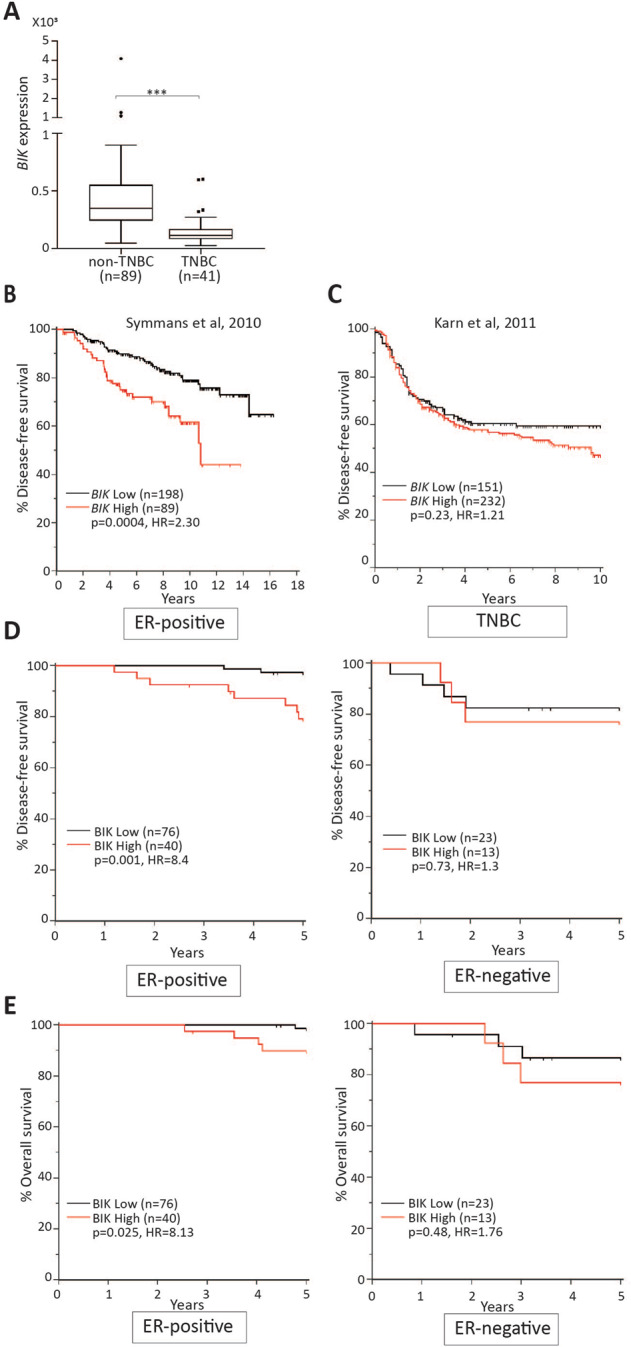
High *BIK* mRNA and protein levels predict poor survival of ER-positive breast cancer patients in multiple datasets. **a** A Tukey box and whisker plot depicting *BIK* gene expression values in non-TNBC and TNBC patients obtained from the publicly available dataset GSE65194. The horizontal line inside the box represents the median. Student′s two-tailed *t*-test was performed to determine statistical significance between the two groups. **b** Kaplan–Meier survival curves depicting disease-free survival outcomes of a total of 287 ER-positive patients stratified into *BIK*-high and -low groups based on *BIK* mRNA levels in tumors. The hazard ratio (HR) value of greater than 1.0 estimates the predicted risk of poor prognosis. *P*-value was calculated using the log-rank test. **c** Kaplan–Meier survival curves depicting disease-free survival outcomes of a total of 383 TNBC patients stratified into *BIK*-high and -low groups based on *BIK* mRNA levels in tumors. **d**, **e** Kaplan–Meier survival curves depicting five-year disease-free or overall survival outcomes of a total 152 of patients stratified in ER-positive antiestrogen treated vs. ER-negative groups. Survival outcomes were calculated based on BIK protein levels in tumor cores. The hazard ratio (HR) value of greater than 1.0 estimates the predicted risk of poor prognosis. *P*-value was calculated using the log-rank test.

To understand the clinical significance of high *BIK* levels in relation to the survival outcomes of breast cancer patients, we examined *BIK* mRNA levels in five publically available datasets^42–48^. Breast cancers can be broadly divided into hormone receptor- and HER2-positive (non-TNBC) or negative (TNBC) categories. Non-TNBCs are either treated with antiestrogens or Herceptin therapy whereas the TNBCs have no targeted therapy and are treated with a combination of chemotherapeutic agents^48,49^. As well, non-TNBC patients have better survival outcomes than the TNBC patients due to the differences in the biology and treatment modalities of the disease^48^. We decided to investigate *BIK* transcript levels in these two groups using publically available gene microarray dataset^45–47^. Intriguingly, we found that non-TNBCs had 3.2-fold increased levels of *BIK* mRNA relative to the TNBCs (Fig. 6a), suggesting relevance to hormonal signaling. Therefore, we next interrogated the prognostic value of *BIK* mRNA levels in ER-positive patients, which represent >80% of all non-TNBC patients. We performed ROC analysis with disease-free survival as a classifier to determine the optimum score cut point. Intriguingly, by utilizing two independent patient cohorts (combined *n* = 345)^42,43^, we discovered that patients with high levels of *BIK* mRNA had an average of 3.7-fold increased risk (*p* < 0.0005) of disease relapse (Fig. 6b and Supplementary Fig. 11A). On the contrary, we found that *BIK* had no predictive value for TNBC patients (Fig. 6c and Supplementary Fig. 11B and C; *p* > 0.05) by analyzing three independent TNBC patient cohorts (combined *n* = 472)^44,45,50^.

In order to interrogate these results at the level of BIK protein, we analyzed whether BIK protein levels predicted relapse in ER-positive breast cancer patients differently than in ER-negative patients (*n* = 152) (Fig. 6D). We determined BIK protein levels by anti-BIK immunohistochemistry followed by ROC analysis to determine the score cut point as described previously^15^. Strikingly, within the ER-positive subtype (*n* = 116), BIK-high patients were 8.4 times more likely to relapse (*p* = 0.001, 95% CI = 2.25–32.0) and were 8.1 times more likely to die from the disease (*p* = 0.025, 95% CI = 1.26–52.5), relative to BIK-low patients (Fig. 6d and e). Intriguingly, 99% of patients with low levels of BIK protein were alive after 5 years. On the other hand, BIK levels did not predict the disease-free (*p* = 0.73, 95% CI = 0.28–6.07) or overall survival (*p* = 0.48, 95% CI = 0.33–9.33) outcomes of the ER-negative patients (Fig. 6d and e).

Thus, BIK predicted recurrence and mortality in ER-positive breast cancer cohorts, suggesting that BIK-mediated sublethal apoptosis may contribute to clinical tumor evolution specifically in this subset of breast cancers.

## Discussion

**Fig. 7:**
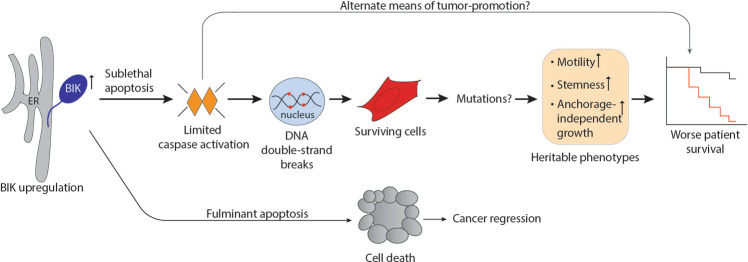
BIK upregulation causes sublethal apoptosis and cancer aggression. Cartoon illustrating the underlying mechanism of tumor-promotion by BIK. ER-localized BIK signals sublethal apoptotic activation in cells leading to limited caspase activation. Activated apoptotic nucleases such as CAD cause DNA double-strand breaks and possible mutations in the surviving cells. These mutations may lead to aggressive cancer phenotypes that are heritable in nature. This mutation-driven tumor evolution combined with other caspase-dependent protumorigenic mechanisms may determine the poor patient prognosis of BIK-high patients.

Cancer treatment aims to minimize residual disease that causes relapse. However, the fate of surviving cells that have escaped cell death after the apoptotic challenge remains largely unknown. In line with this, BIK is well documented to potently induce cell death^24,27,31,51–57^, yet in those studies, 27–65% of cells escaped death and whether these surviving cells developed aggressive phenotypes was unknown. Our study in combination with others^6,10,11,13,58–61^ supports the notion that survivors of apoptosis can actively gain aggressive properties through sublethal caspase signaling in the absence of antiapoptotic upregulation (Fig. 7). In support of this, genetic deletions of proapoptotic molecules, such as BID, PUMA, and caspase-3/7 prevent oncogenesis^59–61^, and high levels of antiapoptotic BCL-2 are associated with favorable patient outcomes in breast, colorectal, and lung cancers^6,7,58,62^. In this context, our study provides novel insight into failed apoptosis triggered by the stress/estrogen deprivation-induced protein BIK and links this adverse association with clinical patient outcome (Fig. 7).

The fate of cells that have been exposed to the long-term apoptotic challenge was not known, and this is highly relevant as breast cancer patients are typically treated with adjuvant hormonal therapy for 2–10 years. Under culture conditions, the majority of BIK-expressing cells not only survive but also proliferate and can form colonies from single cells. At present, the mechanism of cell survival is not clear. Recent studies have identified a process called anastasis wherein cells transiently exposed to apoptotic stimuli show typical morphological features of apoptosis but upon recovery in fresh medium, reattach, and proliferate^63,64^. These surviving cells display aggressive behaviors such as increased motility by upregulating drivers of epithelial to mesenchymal transition (EMT) such as Snail. Thus BIK may cause apoptotic activation followed by recovery in some cells, creating an anastasis-like environment. An interplay between failed apoptosis and anastasis could partly explain the survival of BIK-expressing cells and their acquisition of increased motility after long-term culture. Another possible explanation for the survival of long-term BIK-expressing cells is that BIK stimulates both mild and frank activation of caspases wherein frank caspase activation leads to death of a portion of cell population that is sensitive to BIK-mediated apoptosis. A possibility that this may lead to the natural selection of aggressive-death-resistant cells can not be ruled out at this time. However, given that the majority of BIK-expressing cells show mild caspase activation and limited DNA damage, the ability of these cells to engage cell survival and repair pathways is a likely scenario.

Mutations and genomic instability have a major role in determining cancer heterogeneity. The aggressive phenotypes of BIK-LTC cell lines could be attributed to failed apoptosis-mediated mutagenesis. Caspase and apoptotic nuclease activation (CAD and EndoG) generate mutations and cause carcinogenesis in cell-based models^9,12,65^, although the relevance of these observations in the context of clinical disease was unknown. Interestingly, exome sequencing of 559 ER-positive tumors revealed low- and high mutational load (HML) groups wherein HML tumors were associated with poor prognosis (HR = 2.02)^66^. Considering our results, it would be interesting to determine if the HML tumors have a significantly elevated BIK protein. While mutagenesis driven cancer evolution is a likely scenario for heritable phenotypes that we have observed in vitro, alternative caspase-dependent mechanisms, such as cancer stem-cell enrichment and immune silencing through prostaglandin E2 (PGE2) signaling^67,68^, the competitive outgrowth of treatment-resistant clones^69^, as well as aberrant signaling between apoptotic cells and tumor-associated macrophages that in turn creates a premetastatic niche and facilitates angiogenesis^9,70,71^ may also contribute to cancer promotion in vivo (Fig. 7). Another important consideration is that while our observations support the tumor-promoting role of BIK in cell-based models and associate high BIK levels with poor patient prognosis, a direct causal relationship between BIK elevation, failed apoptosis, and tumor aggression in patient tumors is yet to be established. Further investigations will likely reveal the underlying biology in driving tumor evolution in vivo.

We identified that high levels of BIK are prognostic for ER-positive breast cancer but not for TNBC. Notably, *BIK* mRNA levels are significantly higher in hormonally-driven cancers compared to TNBCs, suggesting a minimum threshold of BIK is required to affect clinical outcomes. We speculate that ER-positive tumors have elevated BIK in response to local deprivation of estrogen, or diminished estrogen signaling, which is known to increase *BIK* mRNA levels^16,18^. Furthermore, ER-positive breast cancer is treated with antiestrogen therapy known to stimulate BIK expression^16,72^. Thus, estrogen-dependent regulation of BIK may trigger sufficient sublethal apoptosis to impact patient outcomes only in the ER-positive subtype. *BIK* mRNA is significantly lower in TNBCs possibly due to the estrogen-independent nature of this subtype. Nevertheless, as seen with our study, ectopic BIK expression in the TNBC cell-line MDA-MB-231 can cause sublethal apoptosis-driven cancer aggression. This observation is crucial given the fact that other tumor-associated stressors, such as hypoxia, DNA damage, and cytokines, can also trigger BIK expression^19^, which may contribute to sublethal apoptosis and tumor evolution in TNBCs as well. This is consistent with our observation that BIK-high TNBC patients show a trend of poor prognosis in three of the four patient cohorts. Thus, irrespective of the nature of the stress, BIK upregulation has the potential to drive tumor aggression in breast cancers. Altogether, our study provides a novel link between weak apoptotic induction and its potential to promote tumor evolution in BIK-high breast cancers.

## Materials and methods

### Lysate preparation from primary flash-frozen tumors

Patient tissue was collected under research ethics board approval (HREB Biomedical, Pro00030345). Primary breast tumors harvested during surgery were flash-frozen and stored at −80 °C until further processing. Tumor tissues were thawed on ice and ground using a Dounce homogenizer in RIPA buffer complete with protease and phosphatase inhibitors. Samples were then spun at maximum speed at 4 °C. The supernatant was obtained and stored at −80 °C. Total protein was quantitated using the BCA protein quantitation kit (Thermo Scientific, USA). 25 μg of total protein was loaded for western blotting.

### Generation of Dox-inducible cell lines

Dox-inducible MCF-7 Tet-On cell line was generated by transfecting MCF-7 cells with pTet-On vector (631018, Clontech, USA) followed by selection with 100 μg/ml G418 (Invitrogen, USA). A single clone was picked, and Tet repressor expression was confirmed using western blot for further experiments. MDA-MB-231 Tet-On cell line was a gift from Dr. Judith Hugh. Dox-inducible BIK-expressing cell lines were produced by cloning of Wt Bik or BikΔBH3 cDNAs into pRetroX-Tight-Pur vector (Clontech, USA) between BamH I and EcoR I restriction sites. Replication-deficient retroviral particles were generated following the manufacturer′s instructions and were used to transduce MCF-7 or MDA-MB-231 cells to generate stable cell lines (632104, Clontech, USA). Briefly, GP2-293 cells were transiently transfected with pRetroX-Tight-pur-Bik and pAmpho plasmids using Lipofectamine 2000 to produce retroviral particles. The supernatant containing viral particles was harvested at 24, 48, and 72 h intervals and MCF-7 or MDA-MB-231 Tet-On cells were transduced with those particles at 24, 48, and 72 h intervals (particles were not pooled) according to the manufacturer′s protocol. Twenty-four hour after transduction, the medium was changed to selection medium containing 1 μg/ml Puromycin (Sigma, USA) and 100 μg/ml G418 for 7 days to eliminate untransduced cells. Stable colonies formed by selected cells were pooled and expanded. Dox-titration to test dose-dependent increase in BIK protein levels was assessed by western blot and immunofluorescence. All cell manipulations up to this point were performed in Tet-free serum (Clontech, USA) and then switched to regular serum (Sigma, USA) after confirmation of lack of leaky BIK expression by western blots. For subsequent experiments, MCF-7 Tet-On BIK and MDA-MB-231 Tet-On BIK cell lines were grown in RPMI + 10% FCS medium after confirming the absence of any leaky expression.

### Western blotting

Cell lysates were prepared in RIPA buffer (50 mM Tris-Cl, 150 mM NaCl, 0.1% SDS, 1% NP-40, 0.5% deoxycholate, and 1 mM EDTA, pH 7.5) supplemented with protease- (11873580001, Roche, USA) and phosphatase inhibitors (04906837001, Roche, USA) and protein concentration was determined using BCA protein assay kit (Thermo Scientific, USA). Twenty microgram of total protein was resolved on 12% SDS-PAGE and blotted on nitrocellulose membranes (Amersham^TM^ Protran^TM^ Premium 0.2 μm NC) followed by probing with the indicated antibodies (Supplementary Table 3) overnight at 4 °C. Next day, HRP-conjugated secondary antibodies (1:5000) (Santa Cruz Biotechnology, BioRad, and Cell signaling technologies, USA) were used followed by signal detection using ECL reagent (Amersham^TM^, UK) and X-ray film (FUJIFILM, Japan). Brightness and contrast adjustments on the images were evenly applied for better visual presentation. Densitometric analysis of the images of scanned blots was performed using ImageJ (1.51j8) program.

### Immunofluorescence

1.5 × 10^5^ cells were grown on glass coverslips (Fisher Scientific, USA) per well in 24-well plates and treated as indicated. For mitochondrion staining, live cells were incubated with 150 nM MitoTracker Red CMXRos (Invitrogen, USA) for 30 min, prior to fixation. Cells were fixed in 4% PFA (Thermo Scientific, USA) at room temperature for 15 min, permeabilized with 0.1% Triton X-100 (Sigma, USA) prepared in 1XPBS and blocked for 1 h with 4% normal goat or donkey serum (Life technologies and Millipore, USA, respectively) depending on the corresponding secondary antibody. Cells were then incubated with appropriate primary antibodies, washed four times with 1XPBS for 5 min each and incubated with 1:250 Alexa Fluor 488 or 555 conjugated secondary antibodies (Life Technologies, USA) along with DAPI (0.25 µg/ml) (Invitrogen, USA). After washing with 1XPBS 3 times, coverslips were mounted on glass slides using ProLong Gold antifade reagent (Molecular Probes, USA). Fluorescent images were captured using either AxioObserver.Z1 microscope (Carl Zeiss, Germany) at ×40 (NA: 1.4) objective using the ZEN2 imaging program. Confocal images were acquired using WaveFx spinning-disk microscope (Quorum Technologies, ON, Canada) using ×20 (NA: 0.85) or ×100 (NA: 1.4) oil immersion objectives using EM-CDD camera (Hamamatsu, Japan) and Volocity software (PerkinElmer, USA) setup on Olympus IX-81 inverted stand (Olympus, Japan). Brightness and contrast adjustments on the images were evenly applied for better visual presentation.

### Cell-viability assay

Cell viability was measured using PI exclusion assay as follows. Briefly, 1.5 × 10^5^ cells were plated per well in 12-well plates followed by a 24 or 48 h Dox stimulation at the indicated Dox concentrations. STS (2.5 µM) was used for 24 h as a positive control for inducing cell death. At the end of treatments, cells were harvested by trypsinization, washed twice with 1XPBS and resuspended in 1XPBS containing 0.5% BSA and 50 µg/ml of propidium iodide (Invitrogen, USA). Flow-cytometric analysis of 10,000 cells for three independent experiments was done using a BD-Accuri flow cytometer using the C6 software.

### Clonogenic survival assays

Thousands cells were plated in 6-well dishes in triplicate and cultured for 12 days (8 days for MDA-MB-231). Fresh growth medium was supplied every 3 days. Colonies were stained with staining solution (0.4% Crystal violet, 4% PFA in 1XPBS), washed, dried overnight, and counted manually.

### Cell-count assays

1 × 10^5^ MCF-7 Tet-On BIK or Empty vector cells were plated per well in 6-well plates in duplicates. The next day, doxycycline treatment was initiated. Fresh medium containing the appropriate concentration of doxycycline was supplemented every 3 days. At the time of harvest, the floating cell population was collected, cells were washed once with 1XPBS, trypsinized and counted using a hemocytometer in duplicates. Trypan blue negative cells were used for cell counts. Three independent experiments were performed.

### TMRE staining and flow cytometry

2 × 10^5^ MDA-MB-231 cells were plated per well in 12-well cell-culture dishes and induced with 250 ng/ml Dox or treated with 2.5 μM staurosporine for 24 h. At the end of the treatments, growth medium containing floaters was collected, adherent cells were washed once with 1 mL 1XPBS and trypsinized with 50 μL trypsin solution for 5 min. Trypsin was neutralized with 1 mL complete growth medium and the cell suspension was spun down at 300 × *g* for 5 min. Resulting cell pellets were resuspended in 500 μL growth medium and a 150 μL aliquot from this was collected in V-bottom plates and centrifuged at 300 × *g* for 5 min. The supernatant was discarded and cell pellets were resuspended in 200 μL complete growth medium containing 0.1 μM Tetramethylrhodamine ethyl ester (TMRE) solution followed by incubation at 37 °C in cell-culture incubator for 15 min. Cells were spun down at 300 × *g* for 5 min, the supernatant discarded and pellets washed once with 1XPBS. The final cell pellets were resuspended in 250 μL of 1XPBS, and passed through a 70 μm nylon sieve to remove any cell clumps. Flow-cytometric analysis was performed on 10,000 cells using the BD-Accuri flow cytometer for at least three independent experiments. Data were analyzed using BD-Accuri c6 program while representative histograms were prepared using Flow Jo (version X) program.

### Active caspase-3/7 staining and flow cytometry

2.5 × 10^5^ MDA-MB-231 cells were plated per well in 12-well cell-culture plates and induced with 250 ng/ml doxycycline or staurosporine for 24 h. Floating cells were collected and adherent cells were trypsinized (50 µL), neutralized with 1 mL growth medium and spun down at 300 × *g* for 5 min. Resulting cell pellets were resuspended in 0.5 mL growth medium and 200 µL of this cell suspension was spun down at 300 × *g* for 5 min in V-bottom plates. The supernatant was discarded and the cell pellets were resuspended in 250 µL growth medium containing 5 µM Cell Event Green (Cat. No. C10723, Invitrogen, USA) reagent for 30 min in cell-culture incubator. Subsequently, cells were passed through a 70 µm nylon sieve to get rid of clumps and flow-cytometric analysis was performed on at least 10,000 cells. Data were analyzed using BD-Accuri c6 program while representative histograms were prepared using Flow Jo (version X) program.

### Fluorescence-activated cell sorting of mild and frank caspase-positive cells

2 × 10^5^ MDA-MB-231 cells were stained with Cell Event Green reagent as described in the Active caspase-3/7 staining and flow cytometry. Thousands cells were sorted directly into 6-well plates containing 2 mL growth medium in duplicates for colony-formation assays while 100,000 cells were collected in a 50/50 mix of 1XPBS and FCS for western blot analysis.

### Quantitation of γH2AX foci and apoptotic nuclei

Foci per nucleus image analysis were performed in MATLAB (MathWorks). For nuclei boundary detection, an Otsu based mask segmentation approach was used^73^, followed by separation of touching nuclei through watersheding. Incomplete nuclei (binary objects touching image edges) were removed and the resultant nuclei boundaries were manually verified. For foci detection, the background image was subtracted from the γH2AX raw images as earlier detailed^73^, using a 10-pixel wide Gaussian. Foci local intensity spots were then detected through iterative filtering from significant coefficients and binarized. The number of foci per nucleus was then computed from the nuclei and foci binary images. Cells with three foci or less were observed in untreated cells and represented basal background levels of γH2AX positivity. To quantitate discrete DNA damage by γH2AX immunofluorescence, we counted % cells with 4–19 foci. Cells with >19 foci were not observed. For apoptotic cells identification, diffuse pan-nuclear γH2AX staining was segmented using the aforementioned nuclei boundary detection method. Resultant binary objects matching nucleus objects were considered apoptotic. A total of 19–20 frames were analyzed for each group.

### Alkaline comet assay

Comet assay was performed as described previously^11,74^. The assay was performed under low light conditions to minimize light-induced DNA damage. Briefly, cells were harvested after appropriate treatments by trypsinization followed by centrifugation (230 × *g* at 4 °C) and resuspended in 1XPBS. Cells were resuspended in 1% low melting point agarose (LMP) at a density of 50 cells/µL; 50 uL of this agarose was put on glass slides (precoated with 1% LMP agarose) and was allowed to bond with the coated agarose for 30 min at 4 °C. Next, agarose-embedded cells were subjected to in situ lysis in lysis buffer (2.5 M NaCl, 100 mM EDTA, 10 mM Trizma base, 1% TX-100, pH 10) at 4 °C for 60 min. Subsequently, nuclear DNA was unwound using freshly prepared alkaline unwinding solution (200 mM NaOH, 1 mM EDTA, pH 8) for 1 h at 4 °C. Subsequently, slides were aligned equidistance from the electrodes and electrophoresis was performed at 300 mA for 30 min in alkaline electrophoresis solution (200 mM NaOH, 1 mM EDTA, pH > 13). Next, the slides were washed with dH_2_O twice followed by 70% ethanol fixation and drying. Electrophoresed DNA was stained with RedSafe DNA stain. Resulting comets were imaged using AxioObserver.Z1 microscope (Carl Zeiss, Germany) at ×10 magnification. %DNA in the tail was calculated using CasPLab comet analysis program (http://casplab.com/download)^75^.

### siRNA transfections

2 × 10^5^ cells were seeded per well in 12-well plates. The next day, 100 nM siRNA oligos were transfected using Lipofectamine 2000 as per manufacturer′s instructions. Knockdown of Dox-induced Bik was performed by transfecting previously reported anti-Bik siRNA 24 h in advance followed by Dox induction for 24 h^27^. CAD silencing was performed in a similar manner. Details of the siRNA oligos used in the study are described in Supplementary Table 2.

### Measurement of ROS levels and DNA damage

Cells were incubated in growth medium containing 2.5 μM CellRox Green reagent (Invitrogen, USA) for 30 min at 37 °C. ROS levels were quantitated by measuring Mean Fluorescence Intensity (MFI) in individual cells using flow-cytometric analysis on a BD-Accuri flow cytometer using the C6 software. TBHP (*tert*-butyl hydroperoxide, 100 μM) (458149, Sigma, USA) was used as positive control. 2.5 mM NAC (N-acetylcysteine) (A9165, Sigma, USA) was used as a ROS scavenger for 30 min prior to TBHP treatment. ROS dependent DNA damage was measured by treating cells with 50 µM H_2_O_2_ for 30 min in the presence or absence of caspase inhibitor z-VAD-fmk (Promega, USA) or ROS scavenger NAC. The DNA damage was confirmed using western blotting with the anti-γH2AX antibody.

### CaspACE staining and flow cytometry

2 × 10^5^ cells/well were seeded in 12-well plates. Twenty-four hour later, cells were treated with the indicated concentrations of dox or staurosporine (2.5 μM) for 24 h. At the end of the treatments, floating cells were collected followed by trypsinization (50 μL) to collect the adherent cell population. Trypsin was neutralized using 1 mL of growth medium. Tubes containing cell suspension were spun at 300 × *g* for 5 min at room temperature. Cell pellets were resuspended in 450 μL growth medium and a 175 μL aliquot was transferred to 96-well V-bottom plates followed by spinning at 300 × *g* for 5 min at room temperature. Resulting cell pellets were resuspended in 200 μL growth medium containing 10 μM CaspAce reagent (#G7461, Promega, USA) or just growth medium for unstained control and incubated in the cell-culture incubator for 1 h. V-bottom plates were spun for 5 min at 300 × *g* and cell pellets were washed twice with 200 μL 0.5% BSA containing 1XPBS followed by final resuspension in 200 μL 0.5% BSA containing 1XPBS. Flow cytometry was performed on a BD-Accuri flow cytometer on the FL-1 channel. For dox-induced cells, at least 10,000 cells while for staurosporine-treated cells 5000 cells were acquired for three independent experiments. Data analysis was performed on FlowJo program_V10.

### Single-cell migration assays

MCF-7 LTC or MDA-MB-231 LTC cells were seeded at a density of 3000 cells/well in 8-well LabTek glass-bottom chambers (#155409, Thermo Fisher Scientific, USA). The following day, live-cell imaging extending up to 24 h was initiated on a ZEN AxioObserver microscope equipped with a heated and humidified stage. Time-lapse images were acquired every 1 h using a 10x objective and Rolera camera. Post-acquisition analysis of single-cell tracks was performed manually on cells that did not undergo cell division using the ImageJ program. Speed was calculated by dividing track length at each time point divided by elapsed time (hours). Rose plots and persistence were computed using the MATLAB program. Persistence was calculated by dividing displacement with track length. Confined and nonconfined classification was determined from individual track mean square displacement curve fit alpha, with alpha values <1 corresponding to confined motion. From three independent experiments, at least 57 and 60 tracks were analyzed in MCF-7 LTC and MDA-MB-231 LTC cells, respectively.

### Collective cell migration assays

2.5 × 10^5^ MCF-7 or MDA-MB-231 LTC cells were seeded in 8-well LabTek glass-bottom chambers (#155409, Thermo Fisher Scientific, USA) to obtain a confluent monolayer the following day. Cells were treated with 10 μg/ml mitomycin C (#M4287, Sigma, USA) for 3 h to block cell proliferation. Subsequently, horizontal scratches were made using a 200 μL pipette tip in one swift motion. Cell debris and mitomycin C were washed away by three gentle washes with growth medium. Subsequently, live-cell imaging extending up to 15 h was initiated on a ZEN AxioObserver microscope equipped with a heated and humidified stage. Time-lapse images were acquired every 1 h using a ×10 objective and Rolera camera. Three different scratch positions per cell line were selected per experiment. Area covered by cells was deducted from the initial area to obtain distance traveled over time using ZEN pro imaging program. At least three independent experiments were performed.

### Soft-agar colony formation

Soft-agar colony formation was done as described previously^11,76^. Briefly, 2500 cells were resuspended in 0.35% agarose (Invitrogen, USA) mixed with complete growth medium and layered on top of a 1% agarose: growth medium mix. Colonies were allowed to form for 7 weeks (6 weeks for MDA-MB-231 LTC) and the agarose layer was kept hydrated by addition of 200 µL of growth medium (RPMI + 10% FCS) every 5 days. Colonies were stained with 0.005% crystal violet (Fisher science education, USA) solution overnight followed by imaging using an EPSON scanner. Soft-agar colonies formed by MDA-MB-231 LTC cells were difficult to visualize on the EPSON scanner. Therefore, representative images were captured using a ZEN AxioObserver microscope on a ×10 objective.

### Mammosphere formation assay

Low cell attachment plates were prepared by incubating 24-well plates with 0.5 ml of 20 mg/ml poly-HEMA (Sigma, USA) solution prepared in 95% EtOH followed by overnight evaporation in the cell-culture hood. EV-LTC-250, BIK-LTC-0, and BIK-LTC-250 single cells were plated in replicate in poly-HEMA coated 24-well plates at 5 cells/mm^3^ suspension (500 µl suspension volume per well) in DMEM/F12 (1:1) supplemented with 20 ng/mL FGF-2 (Sigma, USA), 20 ng/mL EGF (PeproTech), 2% B27 without vitamin A (GIBCO, USA) and 1 × ITS (insulin-transferrin-selenium, GIBCO). 0.5% Methylcellulose (Sigma, USA) was used to prevent cell aggregation, allowing the growth of mammospheres in different z-planes of the medium. Every 3 days, 500 µl of fresh medium was added to each well without removing the old medium. Mammospheres were imaged in brightfield (Zeiss AxioObserver.Z1 Microscope) on days 4, 8, and 12 (on day 28 for MDA-MB-231 LTC). Mammosphere formation efficiency was determined on day 12 (day 28 for MDA-MB-231 LTC) and was calculated from the number of spheres per well, divided by the number of cells plated, multiplied by 100 (to convert it to percentage).

### Mammospheres brightfield images segmentation

Image analyses were performed in MATLAB (MathWorks). To segment mammospheres in brightfield images and determine their areas, a binary gradient image mask of the mammosphere(s) was calculated from a threshold value determined by edge and Sobel operator. Linear gaps in the gradient images were dilated using linear structuring elements, interior holes filled, and resultant mammosphere boundary smoothened by eroding the image twice with a diamond structuring element. Manual inspection of each image was done to ensure correct boundary detection.

### Antiestrogen treatment

Tamoxifen (T5648, Sigma) was dissolved in DMSO at a stock concentration of 1 mM. The experiment was performed under low light conditions to minimize changes in the chirality of Tamoxifen. MCF-7 cells were grown in phenol red-free RPMI + 10% FCS medium and treated with Tamoxifen at the indicated concentrations for 72 h. Cell lysates were prepared in RIPA buffer (50 mM Tris-cl, 150 mM NaCl, 0.1% SDS, 1% NP-40, 0.5% deoxycholate, and 1 mM EDTA, pH 7.5) supplemented with protease- (11873580001, Roche, USA) and phosphatase inhibitors (04906837001, Roche, USA), and BIK expression was confirmed using western blotting.

### Gene Microarray datasets

ER-positive, tamoxifen-treated datasets include GSE17705^43^ and GSE2990^42^. For these datasets, raw data were downloaded, background adjusted and median normalized using the Robust Multi-array Average (RMA) procedure in MATLAB Bioinformatics Toolbox. Original normalized TNBC datasets were downloaded and include GSE31519^50^, GSE33926^44^, and GSE65194^45–47^. To dichotomize patients into *BIK*-low and *BIK*-high groups, an optimum cut point was determined for each dataset by receiver operator characteristic (ROC) curve analysis with disease recurrence as a classification variable following the approach described by^77^ using MedCalc version 15 (Ostend, Belgium). Disease-free survival was calculated by the Kaplan–Meier (KM) analysis. Significant differences between KM curves were measured by the log-rank test.

### Tissue-microarray analysis

Tissue-microarrays (TMA) were prepared as described previously (*n* = 152)^15^. Patient information was collected under research ethics board approval (HREB Biomedical). ER-positive patients were identified by immunohistochemical analysis of tumor biopsies. Immunostaining of the TMAs was performed using an anti-BIK antibody (Santa Cruz Biotechnology, Inc. USA). Scoring of the immunostained TMA was performed in an outcome blinded fashion according to training and guidelines from the study′s breast pathologist. An optimum score cut point was determined by performing ROC curve analysis where disease recurrence was used as a classification variable. Disease-free and overall survival curves were prepared using MedCalc version 15 (Ostend, Belgium). Significant differences between KM curves were measured by the log-rank test.

### Statistical analysis

All bar and line graphs were prepared using GraphPad Prism version 7.03 (GraphPad Software, USA, www.graphpad.com). Statistical significance between two groups was determined using a two-tailed unpaired *t*-test where the alpha was set at 0.05. To determine statistical significance among more than two groups of data, a one-way analysis of variance (ANOVA) was used. Where ANOVA was significant, differences between the two selected groups were analyzed by Sidak′s post-hoc test, and *p*-values were obtained. Following ANOVA where every mean was compared with every other mean, *p*-values were calculated using Tukey′s post-hoc test, whereas where every mean was compared to control mean, *p*-values were calculated using Dunnett′s post-hoc test. Slopes of lines for collective cell migration of LTC cells were calculated by linear regression analysis, and statistical significance was determined using GraphPad Prism version 7.03 (GraphPad Software, USA, www.graphpad.com). (*p < 0.05, **p < 0.01, ***p < 0.001). If no statistical significance was found among the groups analyzed, no asterisks (*) were shown.

## Supplementary information

Supplementary Figure 1

Supplementary Figure 2

Supplementary Figure 3

Supplementary Figure 4

Supplementary Figure 5

Supplementary Figure 6

Supplementary Figure 7

Supplementary Figure 8

Supplementary Figure 9

Supplementary Figure 10

Supplementary Figure 11

Supplementary Figure Legends

Supplementary Table 1

Supplementary Table 2

Supplementary Table 3

## References

[1] Tsujimoto Y, Cossman J, Jaffe E, Croce CM (1985). Involvement of the bcl-2 gene in human follicular lymphoma. Science.

[2] Vaux DL, Cory S, Adams JM (1988). Bcl-2 gene promotes haemopoietic cell survival and cooperates with c-myc to immortalize pre-B cells. Nature.

[3] Billard C (2013). BH3 mimetics: status of the field and new developments. Mol. Cancer Ther..

[4] Delbridge AR, Strasser A (2015). The BCL-2 protein family, BH3-mimetics and cancer therapy. Cell Death Differ..

[5] Roberts AW (2016). Targeting BCL2 with venetoclax in relapsed chronic lymphocytic leukemia. N. Engl. J. Med..

[6] Berardo MD (1998). bcl-2 and apoptosis in lymph node positive breast carcinoma. Cancer.

[7] Vargas-Roig LM (2008). Prognostic value of Bcl-2 in breast cancer patients treated with neoadjuvant anthracycline based chemotherapy. Mol. Oncol..

[8] Neri A (2006). Bcl-2 expression correlates with lymphovascular invasion and long-term prognosis in breast cancer. Breast Cancer Res. Treat..

[9] Ichim G, Tait SW (2016). A fate worse than death: apoptosis as an oncogenic process. Nat. Rev. Cancer.

[10] Ichim G (2015). Limited mitochondrial permeabilization causes DNA damage and genomic instability in the absence of cell death. Mol. Cell.

[11] Liu X (2015). Caspase-3 promotes genetic instability and carcinogenesis. Mol. Cell.

[12] Miles MA, Hawkins CJ (2017). Executioner caspases and CAD are essential for mutagenesis induced by TRAIL or vincristine. Cell Death Dis..

[13] Cartwright, I. M., Liu, X., Zhou, M., Li, F. & Li, C. Y. Essential roles of Caspase-3 in facilitating Myc-induced genetic instability and carcinogenesis. *elife*10.7554/eLife.26371 (2017). 10.7554/eLife.26371 PMC5550274 28691902

[14] Larsen BD, Sorensen CS (2017). The caspase-activated DNase: apoptosis and beyond. FEBS J..

[15] Pandya V (2016). The pro-apoptotic paradox: the BH3-only protein Bcl-2 interacting killer (Bik) is prognostic for unfavorable outcomes in breast cancer. Oncotarget.

[16] Hur J (2004). The Bik BH3-only protein is induced in estrogen-starved and antiestrogen-exposed breast cancer cells and provokes apoptosis. Proc. Natl Acad. Sci. USA.

[17] Hur J (2006). Regulation of expression of BIK proapoptotic protein in human breast cancer cells: p53-dependent induction of BIK mRNA by fulvestrant and proteasomal degradation of BIK protein. Cancer Res..

[18] Coser KR (2003). Global analysis of ligand sensitivity of estrogen inducible and suppressible genes in MCF7/BUS breast cancer cells by DNA microarray. Proc. Natl Acad. Sci. USA.

[19] Chinnadurai G, Vijayalingam S, Rashmi R (2008). BIK, the founding member of the BH3-only family proteins: mechanisms of cell death and role in cancer and pathogenic processes. Oncogene.

[20] Spender LC (2009). TGF-beta induces apoptosis in human B cells by transcriptional regulation of BIK and BCL-XL. Cell Death Differ..

[21] Real PJ (2006). Transcriptional activation of the proapoptotic bik gene by E2F proteins in cancer cells. FEBS Lett..

[22] Ritchie A, Gutierrez O, Fernandez-Luna JL (2009). PAR bZIP-bik is a novel transcriptional pathway that mediates oxidative stress-induced apoptosis in fibroblasts. Cell Death Differ..

[23] Bodet L (2010). BH3-only protein Bik is involved in both apoptosis induction and sensitivity to oxidative stress in multiple myeloma. Br. J. Cancer.

[24] Mebratu YA, Dickey BF, Evans C, Tesfaigzi Y (2008). The BH3-only protein Bik/Blk/Nbk inhibits nuclear translocation of activated ERK1/2 to mediate IFNgamma-induced cell death. J. Cell Biol..

[25] Koong AC (2000). Candidate genes for the hypoxic tumor phenotype. Cancer Res..

[26] Kagawa S (2001). Deficiency of caspase-3 in MCF7 cells blocks Bax-mediated nuclear fragmentation but not cell death. Clin. Cancer Res..

[27] Mathai JP, Germain M, Shore GC (2005). BH3-only BIK regulates BAX,BAK-dependent release of Ca2+ from endoplasmic reticulum stores and mitochondrial apoptosis during stress-induced cell death. J. Biol. Chem..

[28] Germain M, Mathai JP, McBride HM, Shore GC (2005). Endoplasmic reticulum BIK initiates DRP1-regulated remodelling of mitochondrial cristae during apoptosis. EMBO J..

[29] Solier S, Pommier Y (2014). The nuclear gamma-H2AX apoptotic ring: implications for cancers and autoimmune diseases. Cell Mol. Life Sci..

[30] Elangovan B, Chinnadurai G (1997). Functional dissection of the pro-apoptotic protein Bik. Heterodimerization with anti-apoptosis proteins is insufficient for induction of cell death. J. Biol. Chem..

[31] Fu Y, Li J, Lee AS (2007). GRP78/BiP inhibits endoplasmic reticulum BIK and protects human breast cancer cells against estrogen starvation-induced apoptosis. Cancer Res..

[32] Zhou H, Zhang Y, Fu Y, Chan L, Lee AS (2011). Novel mechanism of anti-apoptotic function of 78-kDa glucose-regulated protein (GRP78): endocrine resistance factor in breast cancer, through release of B-cell lymphoma 2 (BCL-2) from BCL-2-interacting killer (BIK). J. Biol. Chem..

[33] Dubrovska A (2012). CXCR4 activation maintains a stem cell population in tamoxifen-resistant breast cancer cells through AhR signalling. Br. J. Cancer.

[34] Morata-Tarifa C (2017). Validation of suitable normalizers for miR expression patterns analysis covering tumour heterogeneity. Sci. Rep..

[35] Zhang L, Xu L, Zhang F, Vlashi E (2017). Doxycycline inhibits the cancer stem cell phenotype and epithelial-to-mesenchymal transition in breast cancer. Cell Cycle.

[36] Vieira AF (2014). P-cadherin signals through the laminin receptor alpha6beta4 integrin to induce stem cell and invasive properties in basal-like breast cancer cells. Oncotarget.

[37] Grimshaw MJ (2008). Mammosphere culture of metastatic breast cancer cells enriches for tumorigenic breast cancer cells. Breast Cancer Res..

[38] Liu Y (2014). Lack of correlation of stem cell markers in breast cancer stem cells. Br. J. Cancer.

[39] Rappa G (2008). Growth of cancer cell lines under stem cell-like conditions has the potential to unveil therapeutic targets. Exp. Cell Res..

[40] Wahler J (2015). Vitamin D compounds reduce mammosphere formation and decrease expression of putative stem cell markers in breast cancer. J. Steroid Biochem. Mol. Biol..

[41] Wang R (2014). Comparison of mammosphere formation from breast cancer cell lines and primary breast tumors. J. Thorac. Dis..

[42] Sotiriou C (2006). Gene expression profiling in breast cancer: understanding the molecular basis of histologic grade to improve prognosis. J. Natl Cancer Inst..

[43] Symmans WF (2010). Genomic index of sensitivity to endocrine therapy for breast cancer. J. Clin. Oncol..

[44] Kuo WH (2012). Molecular characteristics and metastasis predictor genes of triple-negative breast cancer: a clinical study of triple-negative breast carcinomas. PLoS ONE.

[45] Maubant S (2015). Transcriptome analysis of Wnt3a-treated triple-negative breast cancer cells. PLoS ONE.

[46] Maire V (2013). TTK/hMPS1 is an attractive therapeutic target for triple-negative breast cancer. PLoS ONE.

[47] Maire V (2013). Polo-like kinase 1: a potential therapeutic option in combination with conventional chemotherapy for the management of patients with triple-negative breast cancer. Cancer Res..

[48] Carey LA (2006). Race, breast cancer subtypes, and survival in the Carolina Breast Cancer Study. JAMA.

[49] Bertheau P (2013). p53 in breast cancer subtypes and new insights into response to chemotherapy. Breast.

[50] Rody A (2011). A clinically relevant gene signature in triple negative and basal-like breast cancer. Breast Cancer Res..

[51] Zhao X (2008). The endoplasmic reticulum (ER)-target protein Bik induces Hep3B cells apoptosis by the depletion of the ER Ca2+ stores. Mol. Cell Biochem..

[52] Oppermann M (2005). Caspase-independent induction of apoptosis in human melanoma cells by the proapoptotic Bcl-2-related protein Nbk/Bik. Oncogene.

[53] Naumann U (2003). Adenoviral natural born killer gene therapy for malignant glioma. Hum. Gene Ther..

[54] Mebratu YA (2017). Bik reduces hyperplastic cells by increasing Bak and activating DAPk1 to juxtapose ER and mitochondria. Nat. Commun..

[55] Mathai JP, Germain M, Marcellus RC, Shore GC (2002). Induction and endoplasmic reticulum location of BIK/NBK in response to apoptotic signaling by E1A and p53. Oncogene.

[56] Rashmi R, Pillai SG, Vijayalingam S, Ryerse J, Chinnadurai G (2008). BH3-only protein BIK induces caspase-independent cell death with autophagic features in Bcl-2 null cells. Oncogene.

[57] Gillissen B (2007). Mcl-1 determines the Bax dependency of Nbk/Bik-induced apoptosis. J. Cell Biol..

[58] Meterissian SH (2001). Bcl-2 is a useful prognostic marker in Dukes′ B colon cancer. Ann. Surg. Oncol..

[59] Biswas S, Shi Q, Wernick A, Aiello A, Zinkel SS (2013). The loss of the BH3-only Bcl-2 family member Bid delays T-cell leukemogenesis in Atm-/- mice. Cell Death Differ..

[60] Labi V (2010). Apoptosis of leukocytes triggered by acute DNA damage promotes lymphoma formation. Genes Dev..

[61] Michalak EM (2010). Apoptosis-promoted tumorigenesis: gamma-irradiation-induced thymic lymphomagenesis requires Puma-driven leukocyte death. Genes Dev..

[62] Tomita M (2003). Prognostic significance of bcl-2 expression in resected pN2 non-small cell lung cancer. Eur. J. Surg. Oncol..

[63] Sun G (2017). A molecular signature for anastasis, recovery from the brink of apoptotic cell death. J. Cell Biol..

[64] Tang HL (2012). Cell survival, DNA damage, and oncogenic transformation after a transient and reversible apoptotic response. Mol. Biol. Cell.

[65] Lovric MM, Hawkins CJ (2010). TRAIL treatment provokes mutations in surviving cells. Oncogene.

[66] Haricharan S, Bainbridge MN, Scheet P, Brown PH (2014). Somatic mutation load of estrogen receptor-positive breast tumors predicts overall survival: an analysis of genome sequence data. Breast Cancer Res. Treat..

[67] Soteriou D, Fuchs Y (2018). A matter of life and death: stem cell survival in tissue regeneration and tumour formation. Nat. Rev. Cancer.

[68] Huang Q (2011). Caspase 3-mediated stimulation of tumor cell repopulation during cancer radiotherapy. Nat. Med..

[69] Bondar T, Medzhitov R (2010). p53-mediated hematopoietic stem and progenitor cell competition. Cell Stem Cell.

[70] Noy R, Pollard JW (2014). Tumor-associated macrophages: from mechanisms to therapy. Immunity.

[71] Ford CA (2015). Oncogenic properties of apoptotic tumor cells in aggressive B cell lymphoma. Curr. Biol..

[72] Coser KR (2009). Antiestrogen-resistant subclones of MCF-7 human breast cancer cells are derived from a common monoclonal drug-resistant progenitor. Proc. Natl Acad. Sci. USA.

[73] Githaka JM (2016). Ligand-induced growth and compaction of CD36 nanoclusters enriched in Fyn induces Fyn signaling. J. Cell Sci..

[74] Singh NP, McCoy MT, Tice RR, Schneider EL (1988). A simple technique for quantitation of low levels of DNA damage in individual cells. Exp. Cell Res..

[75] Konca K (2003). A cross-platform public domain PC image-analysis program for the comet assay. Mutat. Res..

[76] Borowicz, S. et al. The soft agar colony formation assay. *J. Vis. Exp.*10.3791/51998 (2014). 10.3791/51998 PMC4353381 25408172

[77] DeLong ER, DeLong DM, Clarke-Pearson DL (1988). Comparing the areas under two or more correlated receiver operating characteristic curves: a nonparametric approach. Biometrics.

