# Supplementary figures and images for: BIK drives an aggressive breast cancer phenotype through sublethal apoptosis and predicts poor prognosis of ER-positive breast cancer
Source: Cell Death Dis. 2020 Jun 11;11(6):448. doi: 10.1038/s41419-020-2654-2 (PMC7289861; doi:10.1038/s41419-020-2654-2)

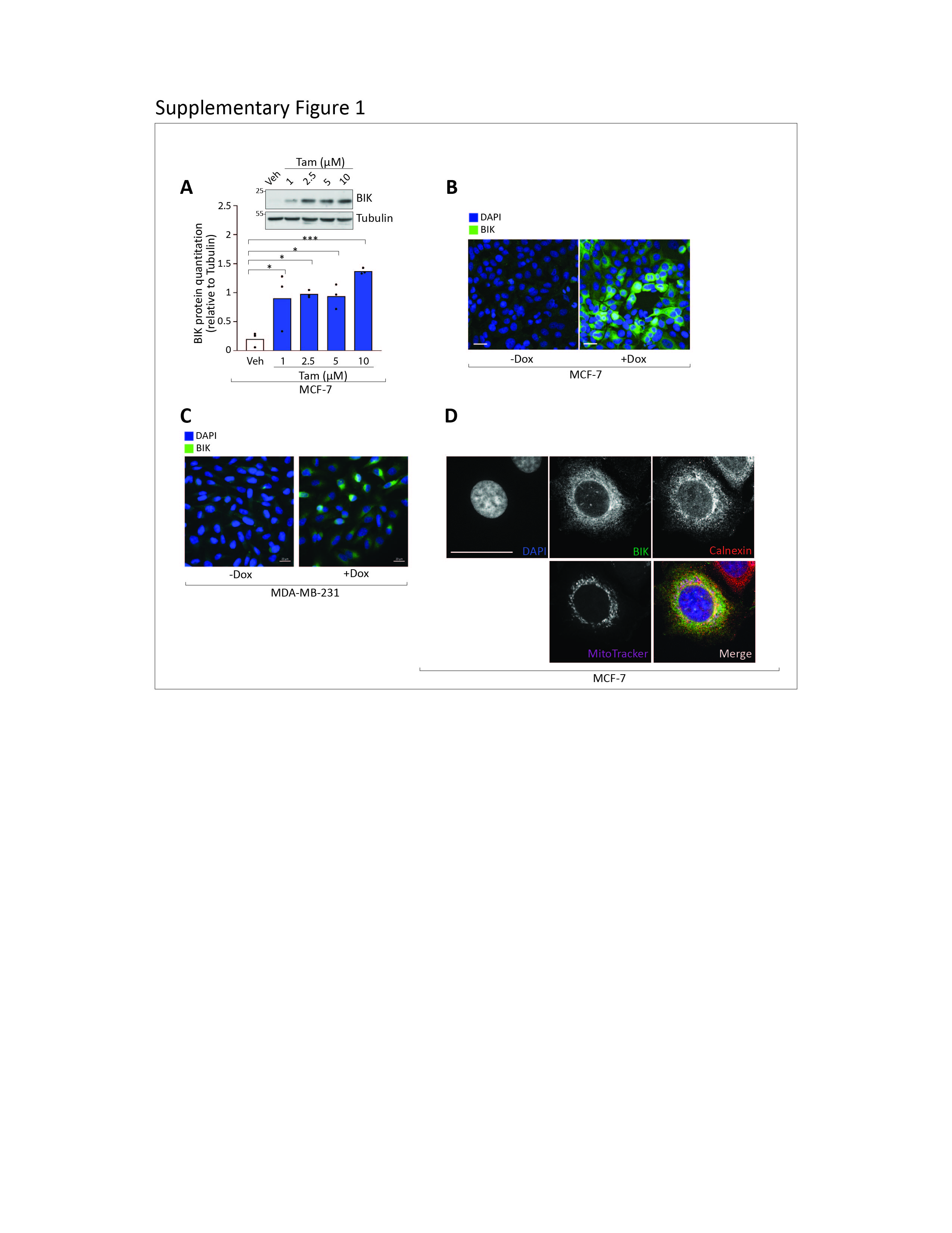

Supplement: Supplementary file 1 — Supplementary Figure 1 [file 41419_2020_2654_MOESM1_ESM.jpg]

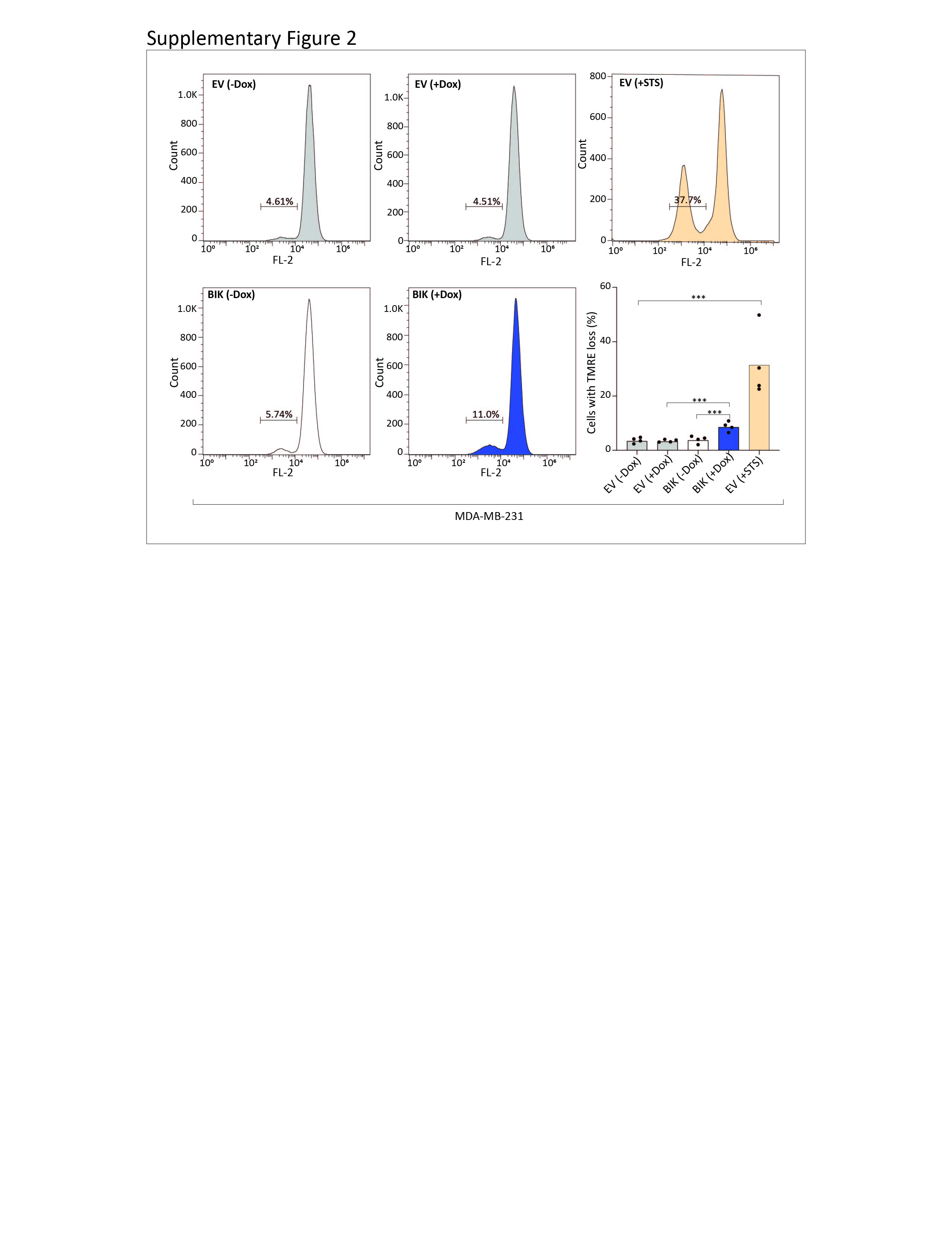

Supplement: Supplementary file 2 — Supplementary Figure 2 [file 41419_2020_2654_MOESM2_ESM.jpg]

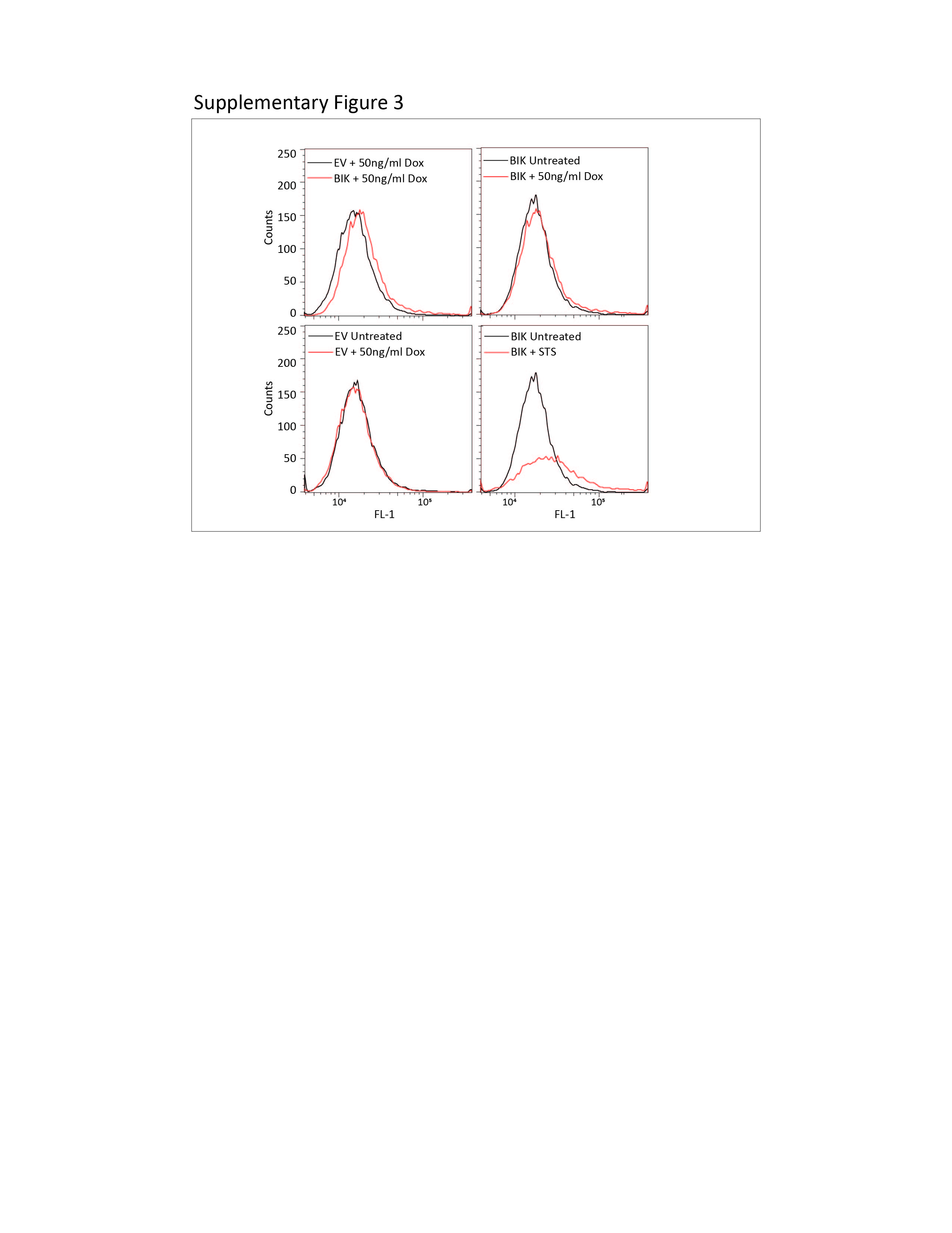

Supplement: Supplementary file 3 — Supplementary Figure 3 [file 41419_2020_2654_MOESM3_ESM.jpg]

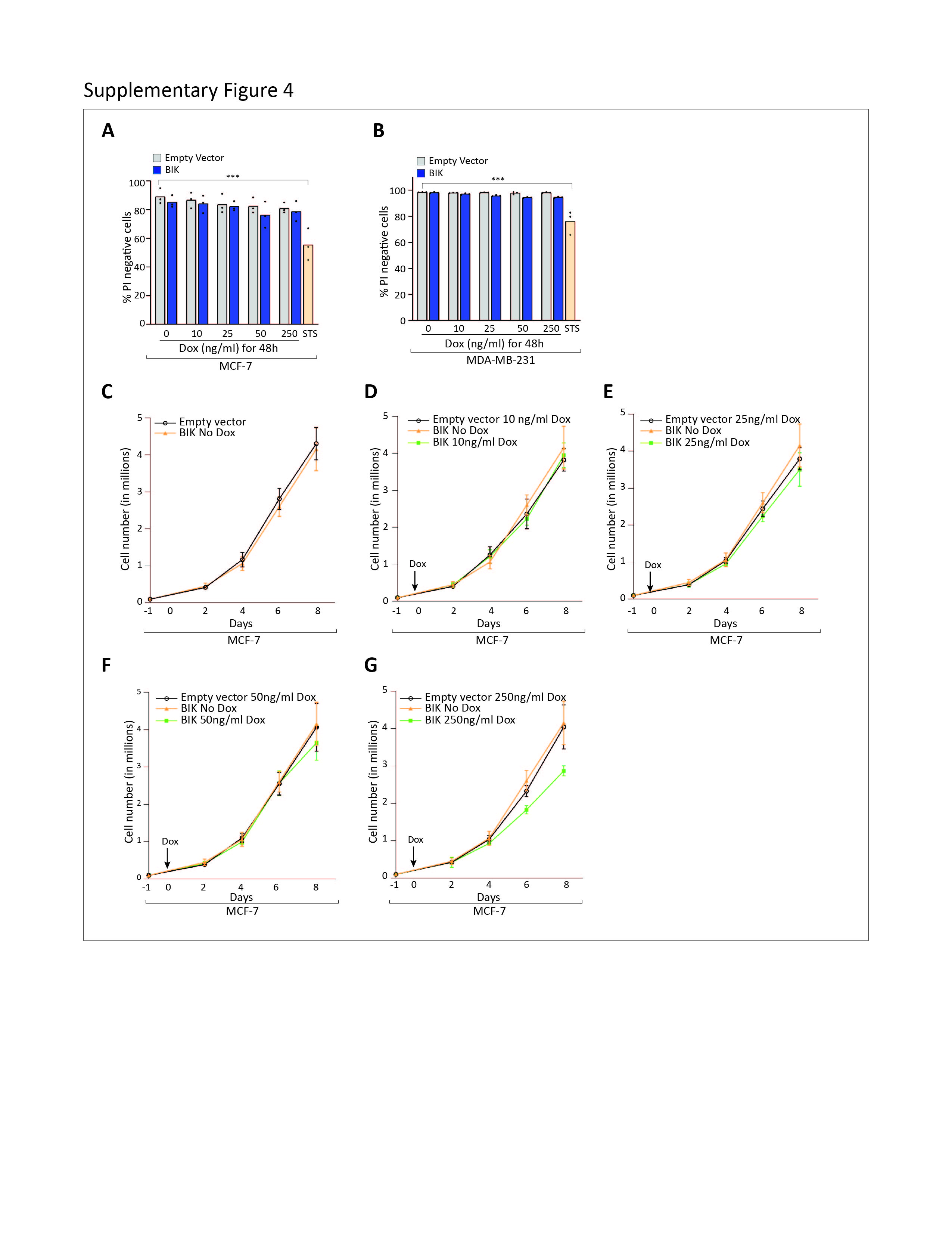

Supplement: Supplementary file 4 — Supplementary Figure 4 [file 41419_2020_2654_MOESM4_ESM.jpg]

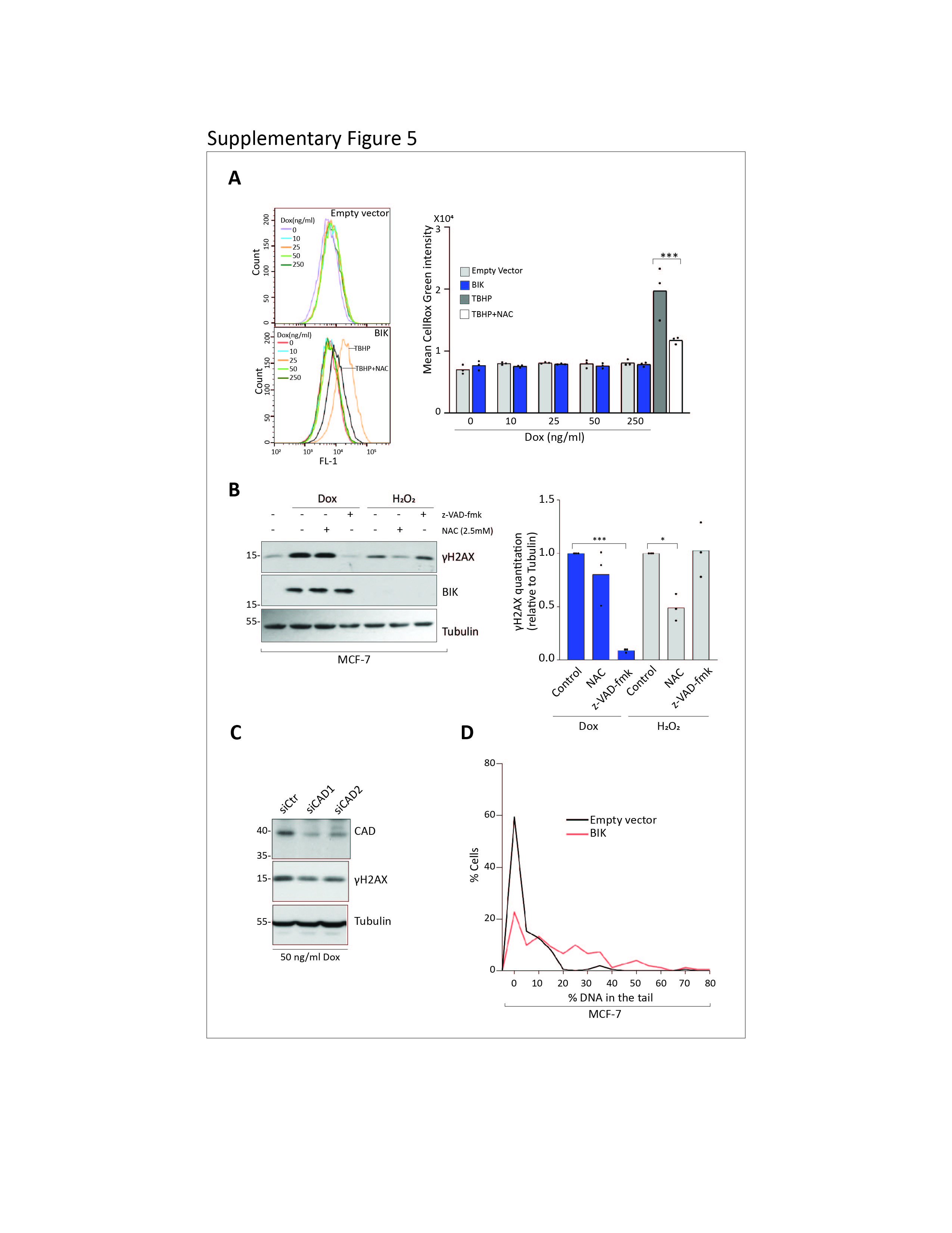

Supplement: Supplementary file 5 — Supplementary Figure 5 [file 41419_2020_2654_MOESM5_ESM.jpg]

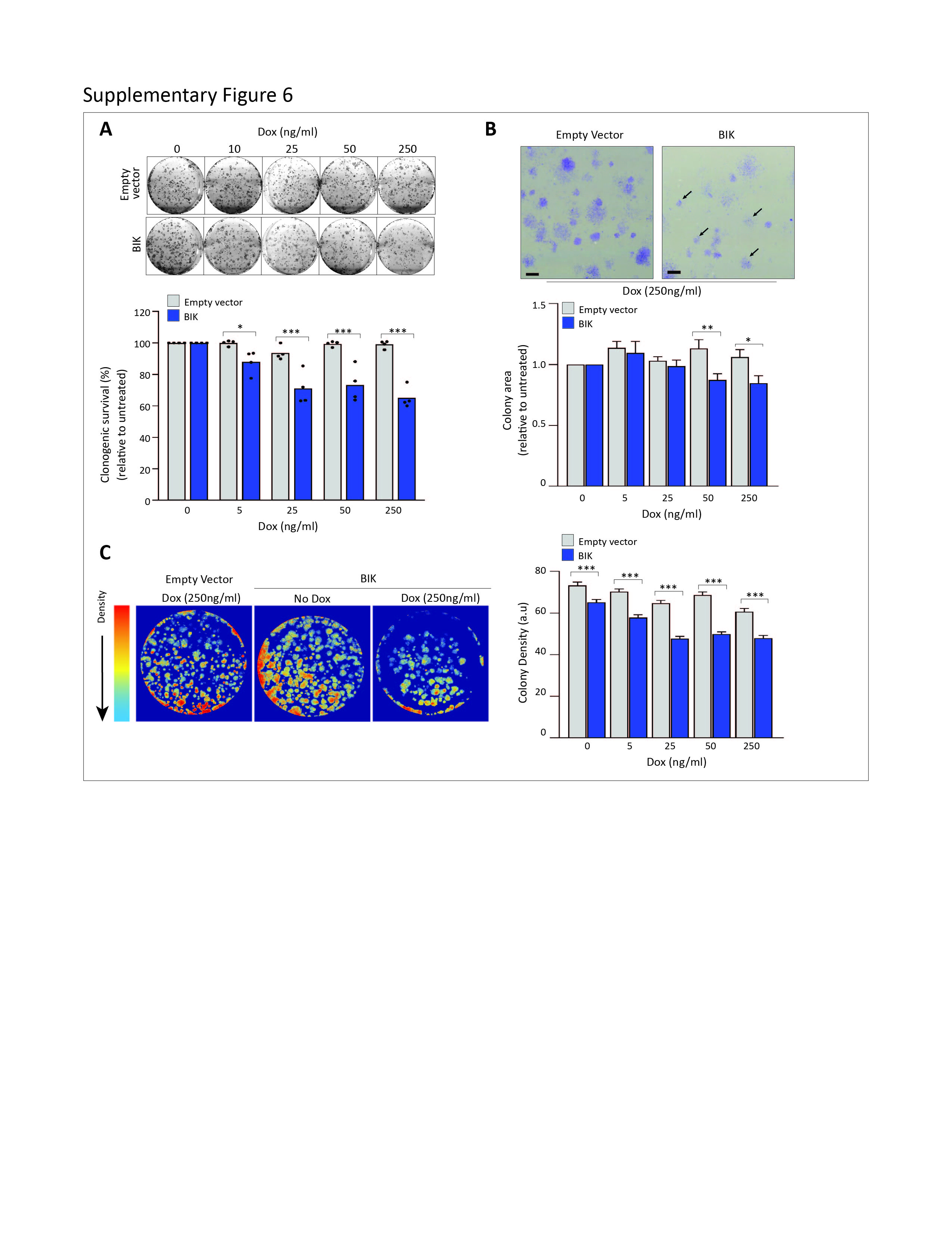

Supplement: Supplementary file 6 — Supplementary Figure 6 [file 41419_2020_2654_MOESM6_ESM.jpg]

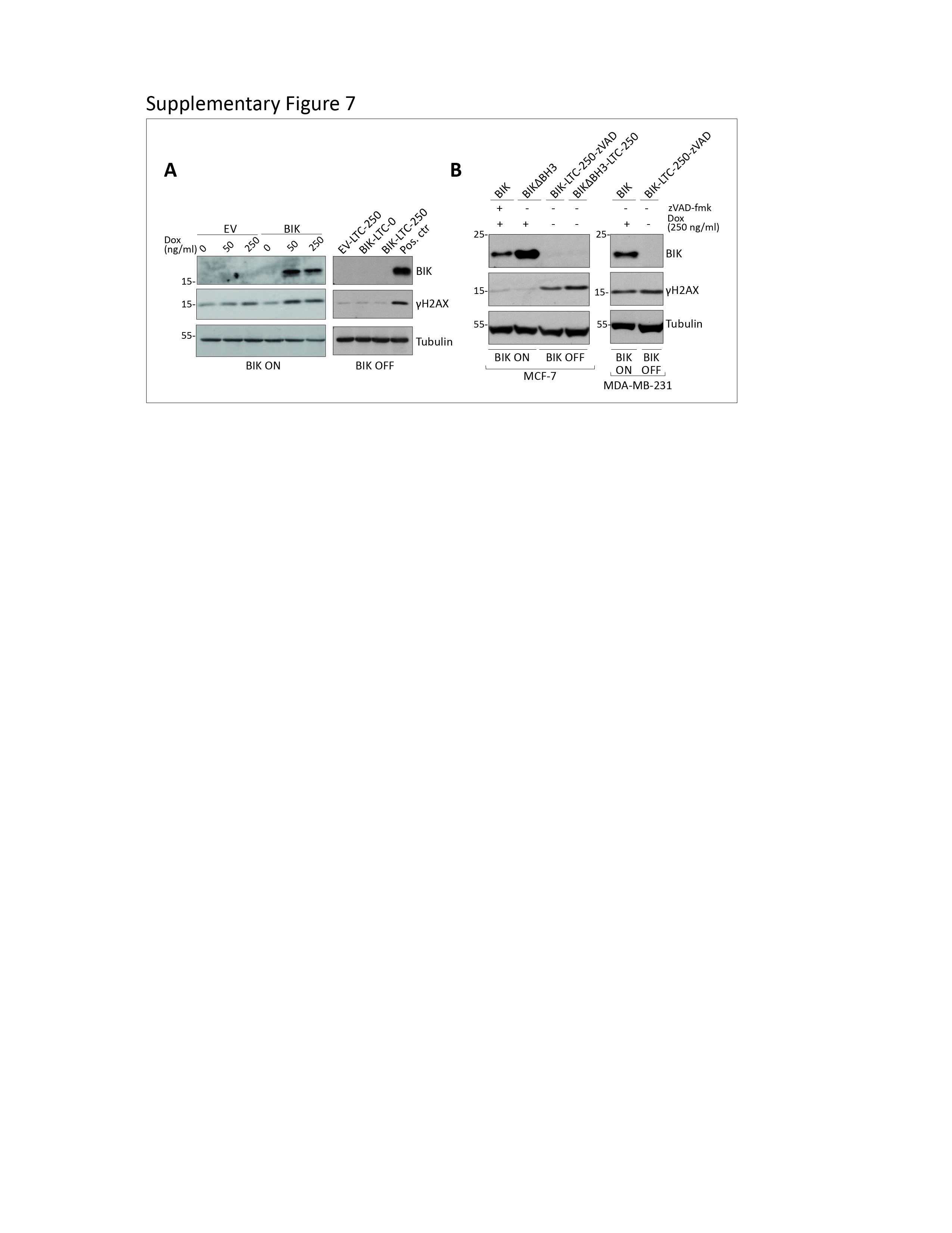

Supplement: Supplementary file 7 — Supplementary Figure 7 [file 41419_2020_2654_MOESM7_ESM.jpg]

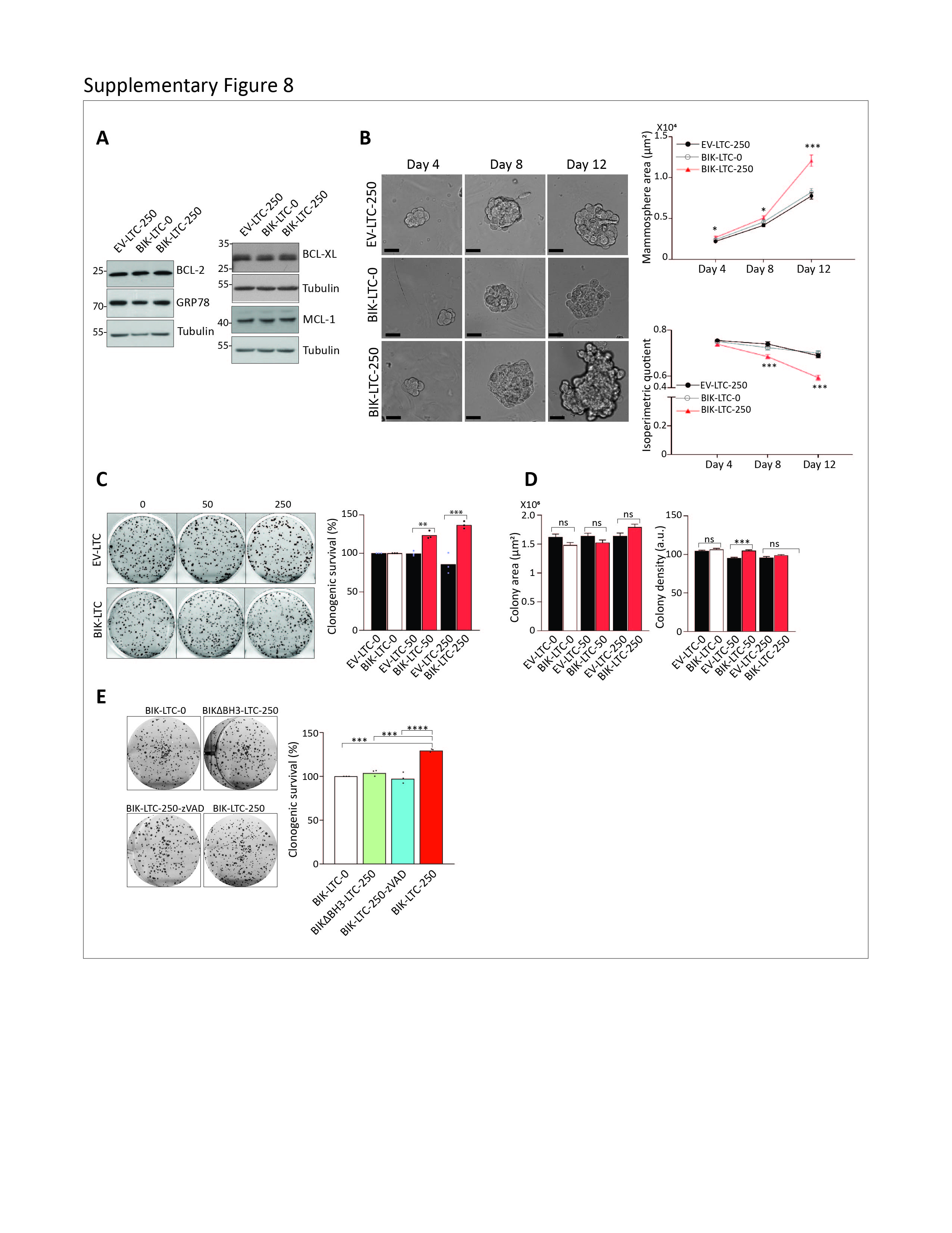

Supplement: Supplementary file 8 — Supplementary Figure 8 [file 41419_2020_2654_MOESM8_ESM.jpg]

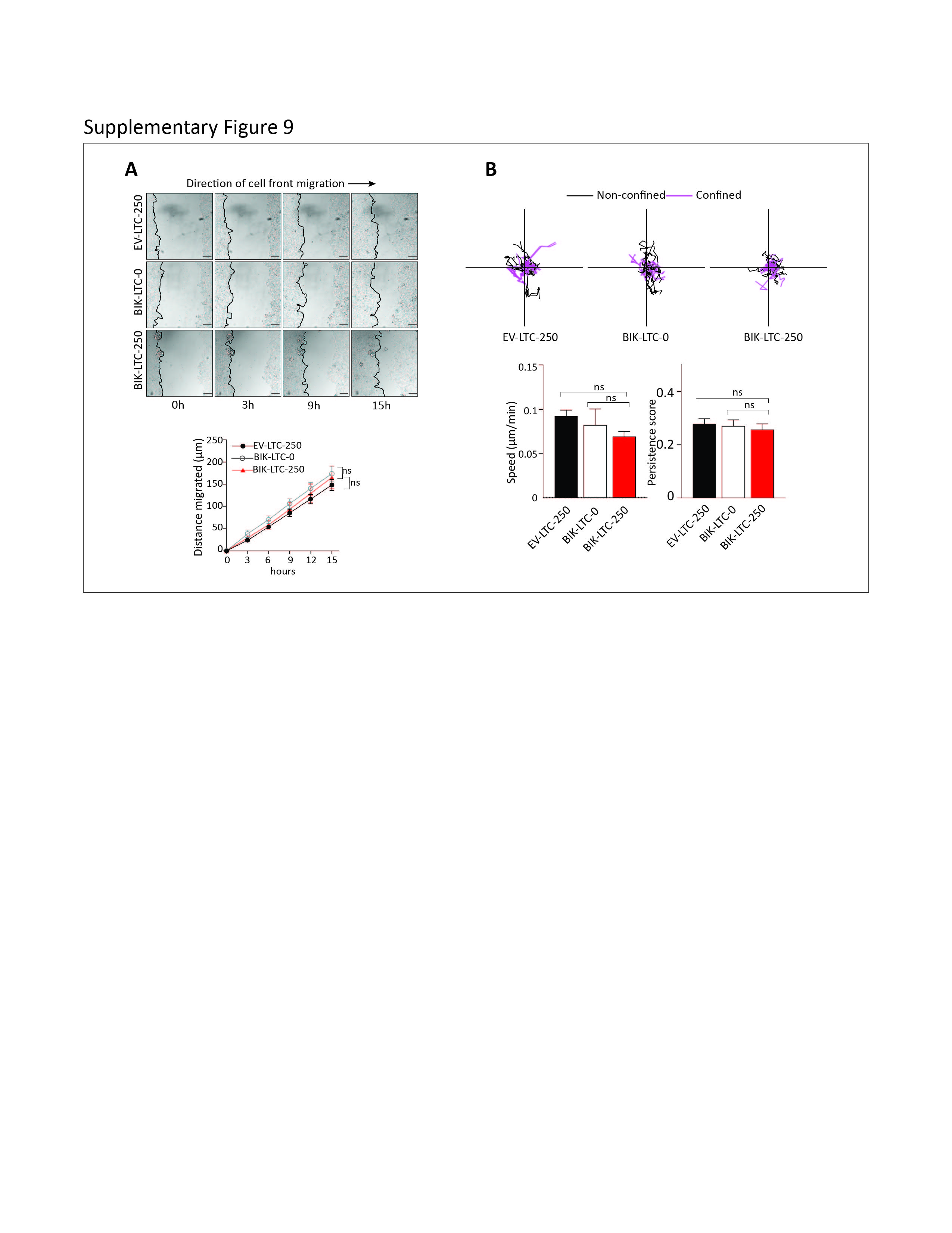

Supplement: Supplementary file 9 — Supplementary Figure 9 [file 41419_2020_2654_MOESM9_ESM.jpg]

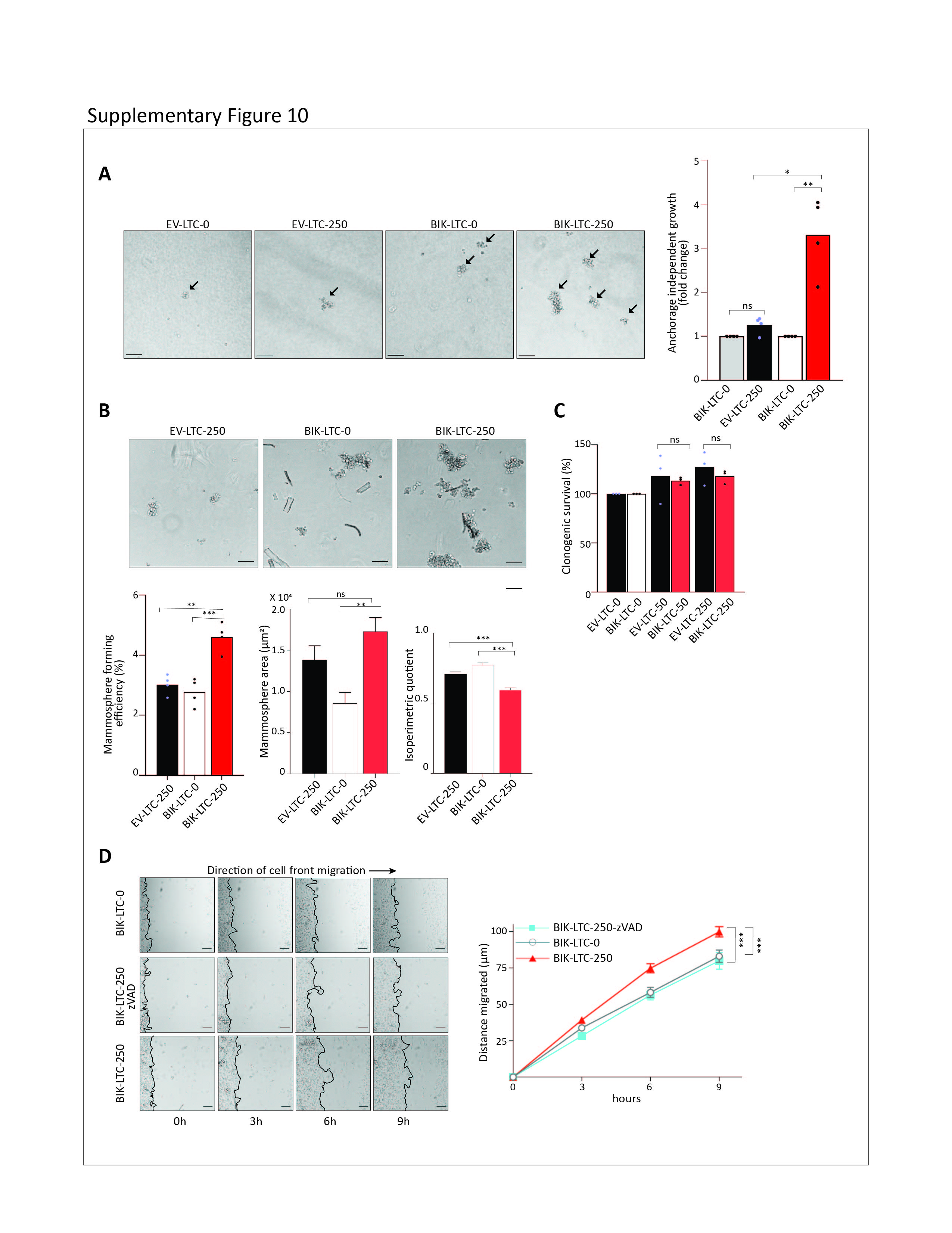

Supplement: Supplementary file 10 — Supplementary Figure 10 [file 41419_2020_2654_MOESM10_ESM.jpg]

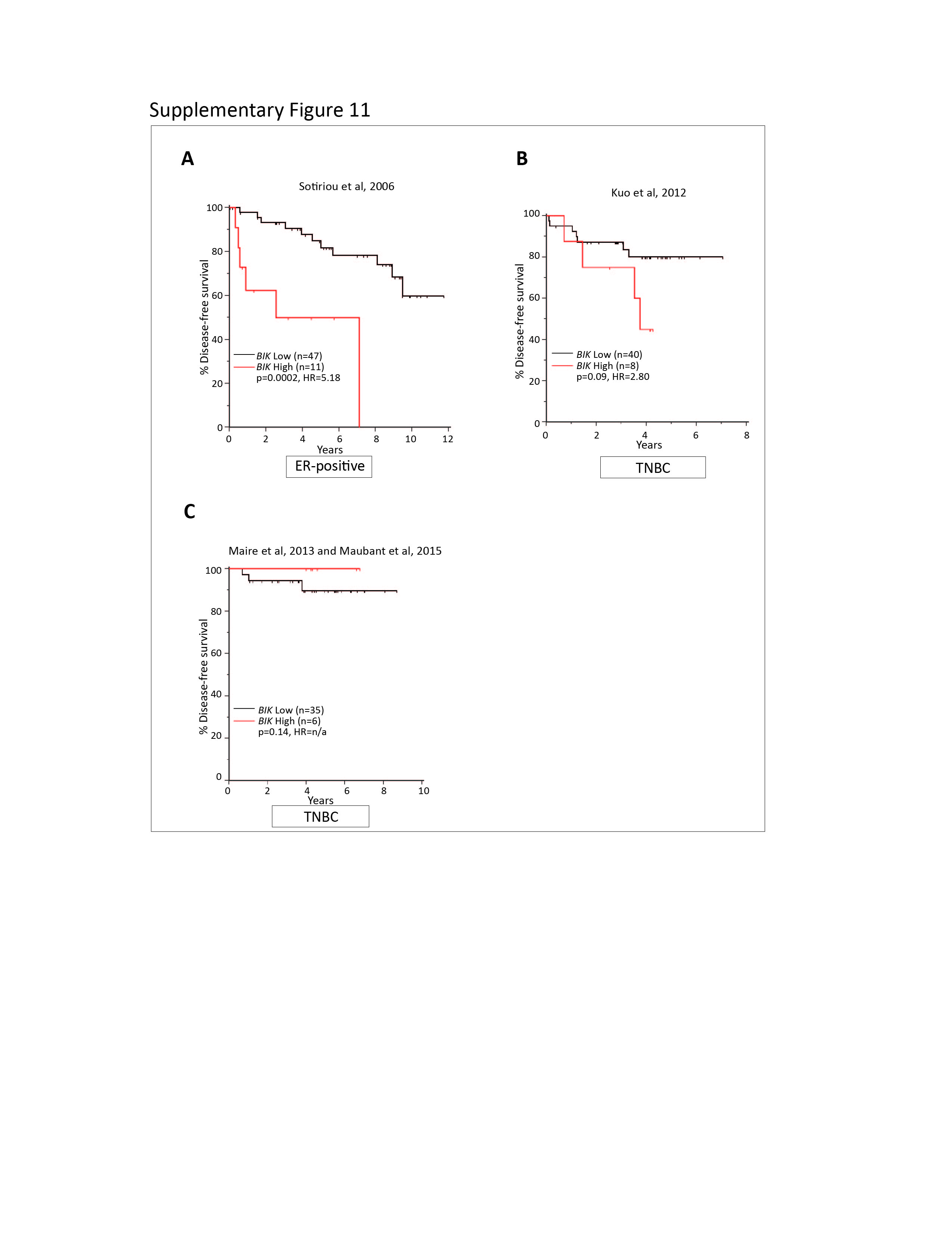

Supplement: Supplementary file 11 — Supplementary Figure 11 [file 41419_2020_2654_MOESM11_ESM.jpg]
