# Supplementary material for: BIK drives an aggressive breast cancer phenotype through sublethal apoptosis and predicts poor prognosis of ER-positive breast cancer
Source: Cell Death Dis. 2020 Jun 11;11(6):448. doi: 10.1038/s41419-020-2654-2 (PMC7289861; doi:10.1038/s41419-020-2654-2)
Supplement: Supplementary file 12 — Supplementary Figure Legends [file 41419_2020_2654_MOESM12_ESM.docx]

**Supplementary Figure Legends**

**Supplementary Figure 1. Tamoxifen-induced BIK expression and immunofluorescence microscopy of BIK expressing cells.**

**A.** Western blot (top) and quantitation (bottom) depicting BIK upregulation with Tamoxifen treatment of MCF-7 cells. Three independent Western blots were used for densitometric quantitation. One-way ANOVA followed by Dunnet’s posthoc test was performed to compute significance among groups. **B.** Immunofluorescence analysis of Dox (250 ng/ml for 24h) stimulated MCF-7 Tet-on cells stained with anti-BIK antibody. Scale bar 50µm. **C.** Immunofluorescence analysis of Dox (250 ng/ml for 24h) stimulated MDA-MB-231 Tet-on cells stained with anti-BIK antibody. Scale bar 20µm. **D**. Immunofluorescence analysis of cells expressing BIK to determine its subcellular localization. MCF-7 Tet-on cells stimulated with 250ng/ml of Dox for 24h were incubated in the MitoTracker-red, followed by 4%PFA fixation and immunostaining with anti-BIK and -calnexin antibodies. Nuclei were stained using DAPI. Images were acquired using a spinning-disk confocal microscope using a 100X oil immersion objective. Scale bar 50µm.

**Supplementary Figure 2. BIK induces mitochondrial potential loss.**

Flow cytometry histograms and bar graph depicting the loss of mitochondrial potential of 24h Dox-stimulated MDA-MB-231 Tet-on cells using TMRE staining followed by flow-cytometric analysis. At least 10 000 cells were acquired for four independent experiments. One-way ANOVA followed by Sidak’s posthoc test was performed to compute significance among groups.

**Supplementary Figure 3. BIK induces caspase activation in MDA-MB-231 cells.**

Flow-cytometry profiles of MDA-MB-231 Tet-on cells stained with CaspACE. Cells were stimulated at the indicated doses of Dox for 24h followed by staining with 10μM of CaspACE (FITC-VAD-fmk) followed by flow-cytometric analysis. Note the rightward shift of the histogram profiles of BIK expressing cells. 2.5μM staurosporine was used as a positive control.

**Supplementary Figure 4. Cell death, proliferation and DNA damage assays of BIK-expressing cells.**

**A and B.** Cell viability of 48h Dox-stimulated MCF-7 and MDA-MB-231 Tet-on cells was respectively determined using flow-cytometric analysis based on their ability to exclude propidium iodide stain. At least 10 000 cells were acquired for three independent experiments. One-way ANOVA followed by Sidak’s posthoc test was performed to compute significance among groups. **C** **to** **G.** MCF-7 Tet-on Empty vector or BIK expressing cells were stimulated at the indicated concentrations of dox, and cell counts were obtained on days 2, 4, 6 and 8. Error bars represent SEM. No statistically significant differences were observed for any of the groups using one-way ANOVA or linear regression analyses.

**Supplementary Figure 5. BIK-mediated DNA damage does not occur through ROS but instead requires caspase activation and CAD in MCF-7 cells.**

**A.** Left: Flow cytometry profiles of ROS measurements by CellRox green reagent for Empty vector or BIK expressing cells at the indicated Dox-stimulation. Right: Bar graph depicting mean Fl-1 intensities indicating ROS levels obtained from the flow-cytometric analysis of at least 10 000 cells from three independent experiments. 50µM TBHP was used as a positive control and 2.5 mM NAC as a ROS scavenger. One-way ANOVA followed by Sidak’s posthoc test was performed to compute significance among groups. **B.** Left: Western blot depicting BIK mediated γH2AX formation independent of ROS but dependent on caspases. Right: Densitometric quantitation of western blots from three independent experiments. One-way ANOVA followed by Sidak’s posthoc test was performed to compute significance among groups. **C.** Western blot depicting knock-down of CAD using two different siRNAs in the presence of 50 ng/ml Dox stimulation of MCF-7 Tet-on BIK cells. **D.** Histogram of comet assay analysis depicting the proportion of cells with various levels of DNA damage. A total of 150 nuclei from three independent experiments were analyzed.

**Supplementary Figure 6. Clonogenic survival assay of MDA-MB-231 cells induced to express BIK.**

**A.** Top: Representative images of clonogenic survival assay performed for Empty vector or BIK expressing MDA-MB-231 Tet-on cells on continuous Dox stimulation at the indicated Dox concentrations over 8 days. Bottom: Bar graph depicting % clonogenic survival relative to untreated. Four independent experiments were performed. One-way ANOVA followed by Sidak’s posthoc test was performed to compute significance among groups. **B.** Top: Representative images of colonies formed by MDA-MB-231 Tet-on Empty vector or BIK expressing cells at 250ng/ml Dox stimulation. Scale bar 1mm. Bottom: Colony area was calculated for at least 350 colonies from each group from four different experiments. Error bars represent SEM. One-way ANOVA followed by Sidak’s posthoc test was performed to compute significance among groups. **C.** Left: Representative images depicting cellular density of colonies formed by MDA-MB-231 Tet-on Empty vector or BIK expressing cells. Red areas indicate high density whereas blue areas indicate low density. At least 350 colonies were analyzed from four independent experiments and measurements were taken. Right: Bar graph depicting quantitation of colony density. Error bars indicate SEM. One-way ANOVA followed by Sidak’s posthoc test was performed to compute significance among groups.

**Supplementary Figure 7.** **MDA-MB-231 and MCF-7 LTC cultures BIK expression and DNA damage.**

**A.** Left: Western blot analysis performed for MDA-MB-231 Tet-on cells after 10 passages in Dox showing the persistence of BIK expression and DNA damage. Right: Western blot analysis showing BIK expression turned off and DNA damage resolved after Dox withdrawal. Cell lysates made from cells expressing BIK were used as a positive control for anti-BIK and -γH2AX antibodies. **B.** Left: Western blot analysis performed for MCF-7 Tet-on cells either expressing Wt BIK (250 ng/ml Dox) and simultaneously treated with 20μM z-VAD-fmk or expressing BIK∆BH3 (250 ng/ml Dox ) at the 10^th^ passage (BIK ON) and after 10 passages (BIK OFF) showing the amount of BIK expression and DNA damage. Right: Western blot analysis performed for MDA-MB-231 Tet-on cells expressing Wt BIK (250 ng/ml Dox) and simultaneously treated with 20μM z-VAD-fmk at the 10^th^ passage (BIK ON) and after 10 passages (BIK OFF) showing the amount of BIK expression and DNA damage.

**Supplementary Figure 8. Aggressiveness characteristics of MCF-7 BIK-LTC cell lines.**

**A**. Western blot analysis of cell lysates prepared from EV-LTC-250, BIK-LTC-0, and BIK-LTC-250 cell lines to test anti-apoptotic proteins upregulation during long-term culture. Blots were probed with antibodies against BCL-2, BCL-XL, MCL-1, and GRP-78. Tubulin was used as a loading control for individual blots. **B.** Left: Representative images depicting the size and shapes of mammospheres formed by MCF-7 LTC cell lines. Scale bar 50µm. Top right: Plot depicting an increase in mammosphere area over time. Error bars represent SEM. Bottom right: Plot depicting the change in circularity over time as measured by the isoperimetric quotient. At least 65 mammospheres from three independent experiments were analyzed. One-way ANOVA followed by Tukey’s posthoc test was performed to compute significance among groups. **C.** Left: Representative images from colony formation assay performed for MCF-7 Tet-on LTC cells. Scale bar 5mm. Right: Quantitation of clonogenic potential relative to untreated controls. Values were obtained from three independent experiments. One-way ANOVA followed by Sidak’s posthoc test was performed to compute significance among groups. **D.** Colony area (left) and colony density (right) was calculated for at least 350 colonies from each group from three different experiments. Error bars represent SEM. One-way ANOVA followed by Sidak’s posthoc test was performed to compute significance among groups. **E.** Left: Representative images from colony formation assay performed for MCF-7 BIK-LTC-0, BIK∆BH3-LTC-250, BIK-LTC-250-zVAD and BIK-LTC-250 cell lines. Right: Quantitation of clonogenic potential relative to untreated control. Values were obtained from three independent experiments. One-way ANOVA followed by Sidak’s posthoc test was performed to compute significance among groups.

**Supplementary Figure 9. Migratory properties of MCF-7 BIK-LTC cell lines.**

**A.** Left: Representative images at the indicated time-points from the collective cell migration assay performed for MCF-7 LTC cells. Right: Quantitation of the movement of cell-front over 15h. A total of 9 positions from three independent experiments was analyzed for each group. Error bars represent SEM. No significant differences were observed with a linear regression analysis of the data. Scale bar 100µm. **B.** Top: Rose plots depicting the spread of cell movements of the MCF-7 LTC cells. Bottom: Speed and persistence scores for MCF-7 LTC cells were calculated by taking the average speed of cells over 24h. At least 60 tracks were analyzed from three independent experiments. Error bars represent SEM. One-way ANOVA followed by Sidak’s posthoc test was performed to compute significance among groups.

**Supplementary Figure 10. Aggressiveness characteristics of MDA-MB-231 BIK-LTC cell lines.**

**A.** Left: Representative images depicting the anchorage-independent growth of MDA-MB-231 LTC cell lines. Right: Quantitation of the fold changes in the number of soft-agar colonies relative to control. Scale bar 100μm. Four independent experiments were performed. One-way ANOVA followed by Sidak’s posthoc test was performed to compute significance among groups. **B.** Top: Representative images from mammosphere formation assay performed with MDA-MB-231 LTC cell lines. Mammosphere forming efficiency (MFE) was calculated after 28 days in culture. Scale bar 100µm. Bottom: Bar graphs depicting quantitation of the MFE, mammosphere area and isoperimetric quotient from four independent experiments. Error bars represent SEM. One-way ANOVA followed by Sidak’s posthoc test was performed to compute significance among groups. **C**. Quantitation of clonogenic potential relative to untreated controls. Values were obtained from three independent experiments. One-way ANOVA followed by Sidak’s posthoc test was performed to compute significance among groups. **D.** Left: Representative images at the indicated time-points from the collective cell migration assay performed for the indicated MDA-MB-231 LTC cell lines. Right: Quantitation of the movement of the cell-front over 9h. A minimum of 9 positions from four independent experiments were analyzed for each group. Error bars represent SEM. Linear regression analysis was performed to calculate differences between groups. Scale bar 100µm.

**Supplementary Figure 11. Kaplan-Meier survival curves of disease-free survivals of ER-positive or TNBC patients based on *BIK* mRNA levels.**

**A.** Kaplan-Meier survival curves depicting disease-free survival outcomes of a total of 58 ER-positive patients stratified into *BIK*-high and -low groups based on *BIK* mRNA levels in tumors. The hazard ratio (HR) value of greater than 1.0 estimates the predicted risk of poor prognosis. P-value was calculated using the log-rank test. **B.** and **C.** Kaplan-Meier survival curves depicting disease-free survival outcomes of a total of 48 (B) and 41 (C) TNBC patients stratified into *BIK*-high and -low groups based on *BIK* mRNA levels in tumors. P-value was calculated using the log-rank test.
