# Supplementary material for: BIK drives an aggressive breast cancer phenotype through sublethal apoptosis and predicts poor prognosis of ER-positive breast cancer
Source: Cell Death Dis. 2020 Jun 11;11(6):448. doi: 10.1038/s41419-020-2654-2 (PMC7289861; doi:10.1038/s41419-020-2654-2)
Supplement: Supplementary file 13 — Supplementary Table 1 [file 41419_2020_2654_MOESM13_ESM.docx]

**Supplementary Table-1 Side by side comparison of the aggressive characteristics of BIK-LTC-250 cell lines derived from MCF-7 and MDA-MB-231 cells.**

|  | **Cell line** | |
| --- | --- | --- |
| **Aggressive property** | MCF-7 BIK-LTC-250 | MDA-MB-231 BIK-LTC-250 |
| Elevated anchorage-independent growth | Yes | Yes |
| Cancer stem cell enrichment | Yes | Yes |
| Higher mammosphere area | Yes | No |
| Reduced mammosphere circularity | Yes | Yes |
| Increased collective cell migration | No | Yes |
| Increased single-cell migration | No | Yes |
| Higher clonogenic potential | Yes | No |
| Increased colony area | No | Yes |
| Lower colony density | No | No |
