# Supplementary material for: BIK drives an aggressive breast cancer phenotype through sublethal apoptosis and predicts poor prognosis of ER-positive breast cancer
Source: Cell Death Dis. 2020 Jun 11;11(6):448. doi: 10.1038/s41419-020-2654-2 (PMC7289861; doi:10.1038/s41419-020-2654-2)
Supplement: Supplementary file 14 — Supplementary Table 2 [file 41419_2020_2654_MOESM14_ESM.docx]

**Supplementary Table-2 Details of siRNA oligos used in the study.**

| **Target gene** | **Oligo name** | **Target Sequence** | **Manufacturer** |
| --- | --- | --- | --- |
| N/A | Control siRNA | Proprietary | Qiagen (SI03650318) |
| Bik | Bik siRNA-1 | AUGCAUGGAGGGCAGUGAC | IDT and Dharmacon |
|  | Bik siRNA-2 | GUCACUGCCCUCCAU GCAU | IDT and Dharmacon |
| CAD | CAD siRNA-1 | GAACCUGGAUCACAUAAUA | Dharmacon |
|  | CAD siRNA-2 | GGACAGCUGCUUAUCAAGA | Dharmacon |
