# Supplementary material for: BIK drives an aggressive breast cancer phenotype through sublethal apoptosis and predicts poor prognosis of ER-positive breast cancer
Source: Cell Death Dis. 2020 Jun 11;11(6):448. doi: 10.1038/s41419-020-2654-2 (PMC7289861; doi:10.1038/s41419-020-2654-2)
Supplement: Supplementary file 15 — Supplementary Table 3 [file 41419_2020_2654_MOESM15_ESM.docx]

**Supplementary Table-3 Details of the antibodies used in the study.**

| **Protein** | **Assay method** | **Cat. No** | **Dilution medium** | **Dilution** |
| --- | --- | --- | --- | --- |
| Phspho Ser139-H2AX | IF | CST-8718S | 4%NDS | 1:500 |
|  | IB |  | 5% BSA | 1:3000 |
| BIK | IHC | SC-1710 | 3% BSA | 1:300 |
|  | IF |  | 4% NDS | 1:500 |
|  | IB |  | 5% Milk | 1:500 |
| Caspase-7 (full length) | IB | CST-9492 | 5% Milk | 1:1000 |
| Caspase-7 (cleaved) | IB | CST-9491 | 5% Milk | 1:1000 |
| CAD | IB | ProScie-2107 | 5% Milk | 1:200 |
| Calnexin | IF | ^78^ | 4% NDS | 1:250 |
| GRP78 | IB | Abcam-Ab21685 | 5% Milk | 1:2000 |
| BCL-2 | IB | CST-2872S | 5% Milk | 1:2000 |
| BCL-XL | IB | CST-2762S | 5% Milk | 1:1000 |
| MCL-1 | IB | Sigma-M8434 | 5% Milk | 1:1000 |
| Tubulin | IB | Sigma-5168 | 5% milk | 1:100,000 |
